# Neural Representations of Covert Attention across Saccades: Comparing Pattern Similarity to Shifting and Holding Attention during Fixation

**DOI:** 10.1523/ENEURO.0186-20.2021

**Published:** 2021-03-05

**Authors:** Xiaoli Zhang, Julie D. Golomb

**Affiliations:** Department of Psychology, The Ohio State University, Columbus, OH 43210

**Keywords:** covert attention shifts, fMRI, reference frames, representational similarity, saccades

## Abstract

We can focus visuospatial attention by covertly attending to relevant locations, moving our eyes, or both simultaneously. How does shifting versus holding covert attention during fixation compare with maintaining covert attention across saccades? We acquired human fMRI data during a combined saccade and covert attention task. On Eyes-fixed trials, participants either held attention at the same initial location (“hold attention”) or shifted attention to another location midway through the trial (“shift attention”). On Eyes-move trials, participants made a saccade midway through the trial, while maintaining attention in one of two reference frames: the “retinotopic attention” condition involved holding attention at a fixation-relative location but shifting to a different screen-centered location, whereas the “spatiotopic attention” condition involved holding attention on the same screen-centered location but shifting relative to fixation. We localized the brain network sensitive to attention shifts (shift > hold attention), and used multivoxel pattern time course (MVPTC) analyses to investigate the patterns of brain activity for spatiotopic and retinotopic attention across saccades. In the attention shift network, we found transient information about both whether covert shifts were made and whether saccades were executed. Moreover, in this network, both retinotopic and spatiotopic conditions were represented more similarly to shifting than to holding covert attention. An exploratory searchlight analysis revealed additional regions where spatiotopic was relatively more similar to shifting and retinotopic more to holding. Thus, maintaining retinotopic and spatiotopic attention across saccades may involve different types of updating that vary in similarity to covert attention “hold” and “shift” signals across different regions.

## Significance Statement

To our knowledge, this study is the first attempt to directly compare human brain activity patterns of covert attention (to a peripheral spatial location) across saccades and during fixation. We applied fMRI multivoxel pattern time course (MVPTC) analyses to capture the dynamic changes of activity patterns, with specific focus on the critical time points related to attention shifts and saccades. Our findings indicate that both retinotopic and spatiotopic attention across saccades produce patterns of activation similar to “shifting” attention in the brain, although both tasks could be interpreted as “holding” attention by the participant. The results offer a novel perspective to understand how the brain processes and updates spatial information under different circumstances to fit the needs of various cognitive tasks.

## Introduction

We live in a world with an abundance of visual information, but we have limited visual acuity and cognitive resources. To process visual information across various locations with high sensitivity as needed by daily tasks, we can perform functions like shifting attention allocation covertly or making eye movements. In daily life, covert attention shifts and saccades are often directed to the same to-be-attended location. But we can also covertly attend one location while saccading elsewhere, and the neural mechanisms underlying this case are considerably less explored.

When the eyes are at a stable fixation, covert shifts of attention are often associated with activation in the frontoparietal network (Chica et al., 2013). Specifically, medial superior parietal lobule (SPL) is activated when covert attention is shifted spatially (Yantis et al., 2002; Gmeindl et al., 2016), between space and feature dimensions (Greenberg et al., 2010), between visual and auditory modalities (Shomstein and Yantis, 2004), and between spatial and nonspatial modalities (Shomstein and Yantis, 2006), suggesting the presence of a general mechanism that mediates shifts of attention.

A number of studies comparing covert attention shifts with overt attention shifts (saccades) further show that these two functions share overlapping brain areas, including intraparietal sulcus (IPS), SPL, and frontal regions like precentral sulcus/gyrus (Corbetta et al., 1998; Perry and Zeki, 2000; Beauchamp et al., 2001; de Haan et al., 2008). In these neuroimaging studies, a common paradigm is for participants to either shift attention (covert shifts) or make a saccade (overt shifts) between the current fixation point and a target location, with the brain activation in these conditions each contrasted with a baseline condition where no shift happened.

These neuroimaging studies, together with behavioral evidence, suggest a tight coupling between covert spatial attention and eye movements. Covert attentional orientation is an important step preceding saccade execution (Kowler et al., 1995; Peterson et al., 2004). The premotor theory of attention even claims that covert attention simply reflects the central programming of eye movements, just without actual saccade execution (Rizzolatti et al., 1987). However, this theory remains controversial, especially regarding independence between endogenous attention and motor preparation (Smith and Schenk, 2012), and covert spatial attention and saccade target locations can be dissociated in several paradigms, such as anti-saccade tasks (Juan et al., 2004; Smith and Schenk, 2007) and attention in different reference frames, as below.

When attention is allocated to a separate location from the saccade target, the eye movement introduces a discrepancy between retinotopic (eye-centered) and non-retinotopic (e.g., spatiotopic/world-centered) reference frames. Although the spatiotopic reference frame feels more relevant for most behaviors, visual processing starts on our retina in retinotopic coordinates. Behavioral and neural evidence shows that we can allocate attention in both retinotopic and spatiotopic reference frames, though it is debated which is more dominant and whether they differ by brain region (Melcher and Morrone, 2003; Golomb et al., 2008; Crespi et al., 2011; Golomb and Kanwisher, 2012a,b; Satel et al., 2012; Turi and Burr, 2012; Zimmermann et al., 2013; Fabius et al., 2016; Fairhall et al., 2017; Shafer-Skelton and Golomb, 2018).

This ambiguity raises important questions about how our brain processes covert attention across saccades. For example, maintaining covert attention at a stable peripheral real-world location across a saccade (i.e., spatiotopic attention) would be akin to holding attention in spatiotopic coordinates, but shifting attention in retinotopic coordinates. Here, we take a novel approach to understanding the relationship between covert attention and saccades by comparing the neural patterns associated with retinotopic and spatiotopic attention across saccades to holding or shifting covert attention during fixation. We hypothesized that activation patterns should reveal whether maintaining retinotopic or spatiotopic attention is represented more like a stable hold of attention, in functionally relevant regions such as the attention shift network.

We acquired fMRI data during a combined saccade and covert attention task, with four critical conditions. On Eyes-fixed trials, participants either held attention at the same initial peripheral location (“hold attention”) or shifted attention to a different location midway through the trial (“shift attention”). On Eyes-move trials, participants made a saccade midway through the trial half of the time, while covertly maintaining either “spatiotopic attention” (hold relative-to-screen, shift relative-to-eyes) or “retinotopic attention” (hold relative-to-eyes, shift relative-to-screen). We used multivoxel pattern time course (MVPTC) analyses to compare whether patterns of brain activity for spatiotopic and retinotopic conditions were more similar to shifting or to holding attention, both in our a priori regions of interest (ROIs), as well as through an exploratory whole-brain searchlight analysis.

## Materials and Methods

### Participants

Twelve right-handed subjects participated in the study (seven females, five males, mean age 19.08, range 18–25). An additional left-handed subject was also scanned inadvertently, but the data were not included in our analyses. All subjects reported normal or corrected-to-normal vision. They were prescreened for MRI eligibility, and they gave informed consent. The study protocol was approved by the Ohio State University Biomedical Sciences Institutional Review Board.

### Stimuli and task

**Figure 1.**
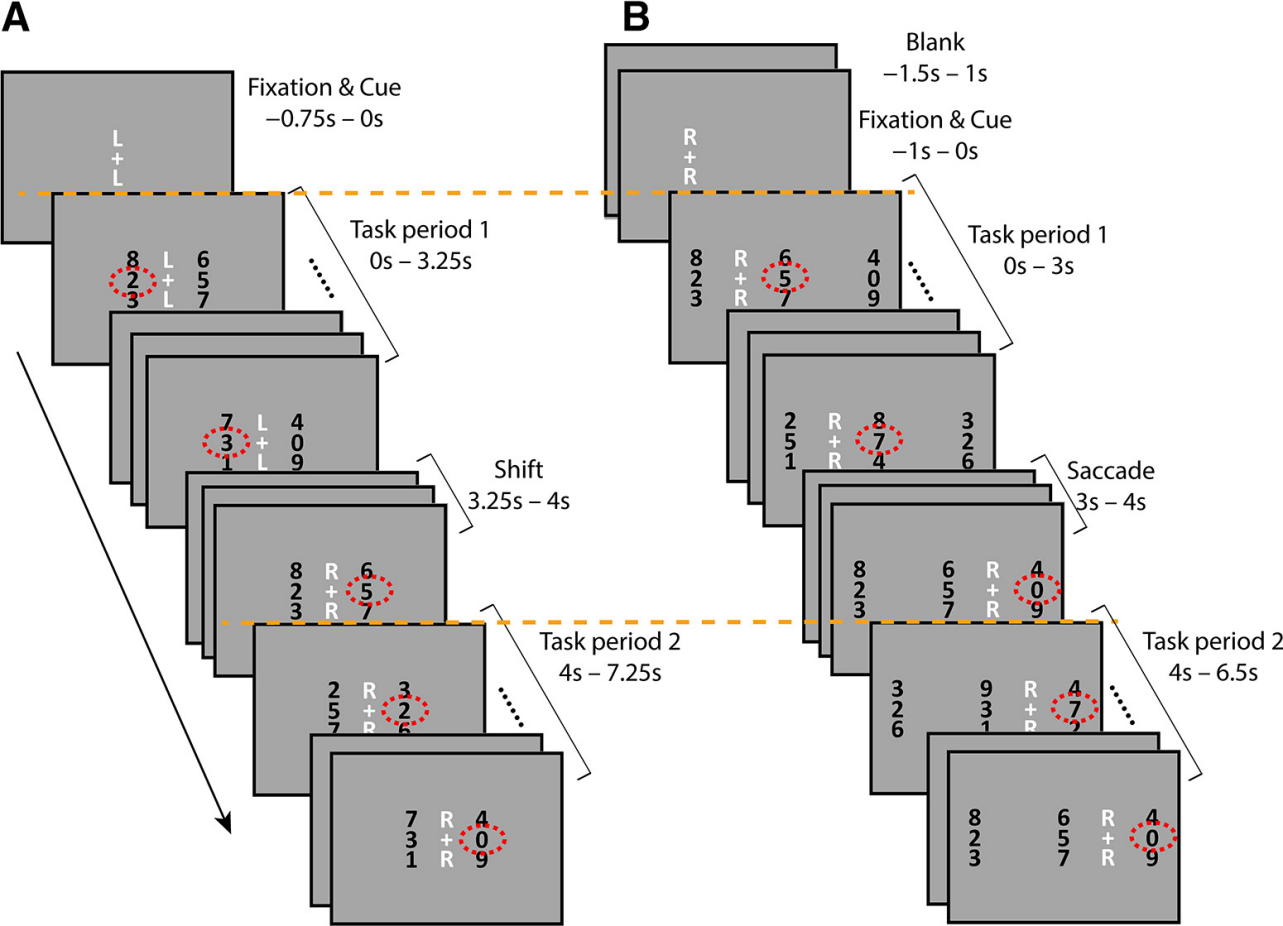
Paradigms of the Eyes-fixed and Eyes-move tasks. ***A***, An example of an Eyes-Fixed, Shift-attention trial, where covert attention is shifted from the left stream to the right stream; the letter cues “L” and “R” above and below the fixation cross indicate “left” and “right.” ***B***, An example of an Eyes-move, maintain Retinotopic-attention trial, where covert attention is maintained on the stream located to the right of fixation across the saccade; here the letter cues L and R indicate “left of fixation” and “right of fixation,” and “C” would indicate “center of screen” for maintain Spatiotopic-attention trials (see Fig. 2 for examples). Red dotted circles (not shown in the actual experiment) indicate the digit stream that participants should attend to according to the letter cue. Time 0 s is taken as the onset of each trial, and orange dotted lines are to show that the onsets of task periods 1 and 2 were synced with scanner pulse in both Eyes-fixed and Eyes-move tasks.

The paradigm is shown in Figure 1. Eyes-fixed and Eyes-move tasks were done in separate runs.

In the Eyes-fixed task (Fig. 1*A*), subjects fixated their eyes at the fixation cross at the screen center. A letter cue appeared above and below the fixation to indicate the location to be covertly attended (L for left of fixation, R for right of fixation). The stimuli were rapid serial visual presentation (RSVP) streams of random digits (each frame of digits presented for 250 ms without gap). Two columns of RSVP streams were located 2.5° to the left and right of the fixation cross, respectively. In each column, the middle stream was the target stream and the upper and lower streams were the flanker streams. Subjects were instructed to attend to the cued side and press the button when they saw a target (the number 5) in the target stream.

**Figure 2.**
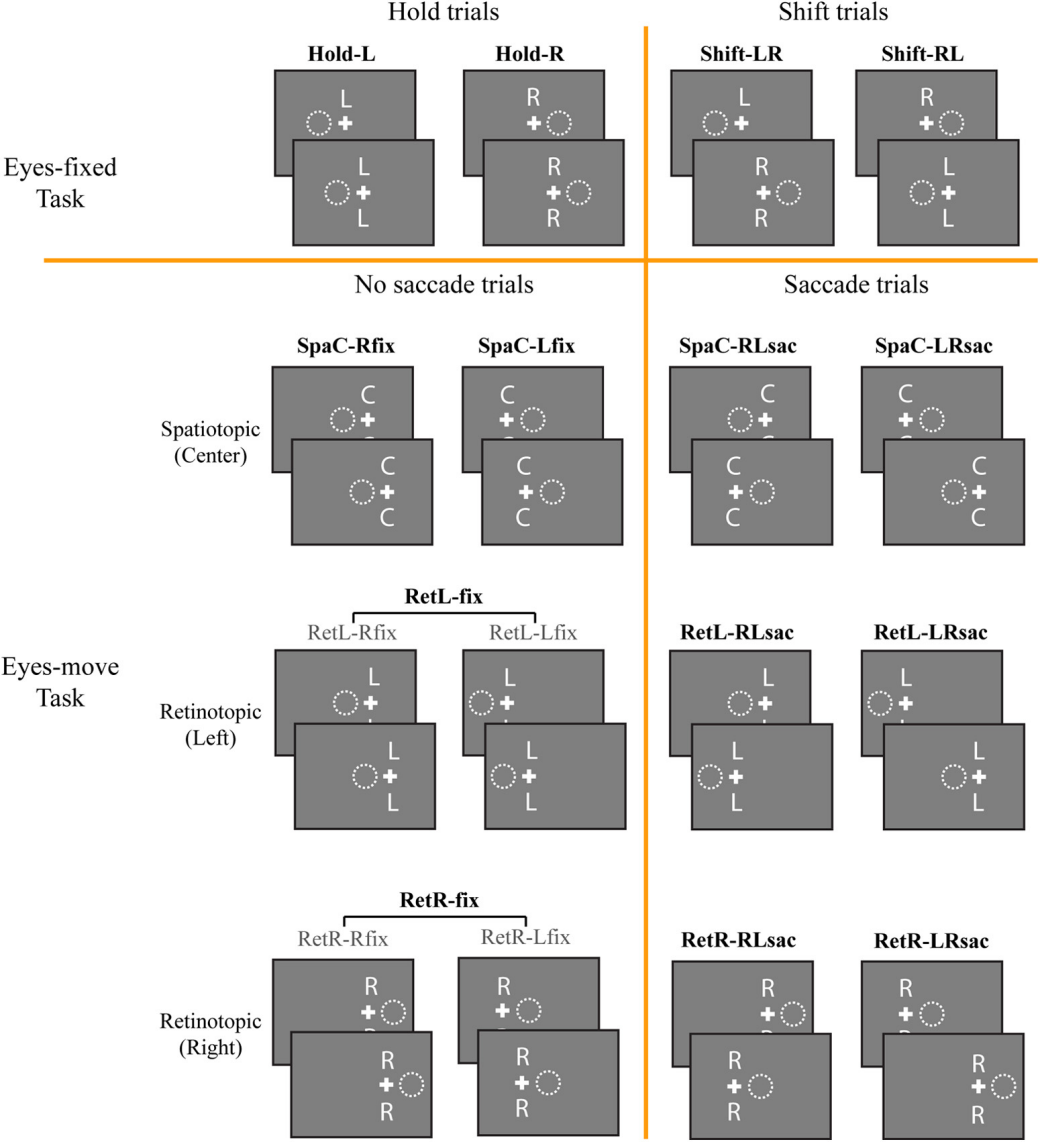
Diagrams of all conditions. Each condition is separated into the first half (before shift/saccade) and the second half (after shift/saccade), shown as the top and bottom panel for each condition. White crosses indicate the fixation location, and white dotted circles indicate the attention location on the screen, corresponding to the letter cues above and below the fixation. Note that in our analyses, we did not separate the left and right fixation for retinotopic no-saccade conditions; that is, only the bolded conditions were included in the GLMs.

Each trial lasted 8 s. The fixation and letter cue alone were presented for 0.75 s before the onset of the RSVP streams. On half of the trials, the letter cue changed (e.g., from L to R) midway through the trial (always 3.25 s after the onset of the RSVP streams), cueing participants to shift their covert attention to the other side and monitor for the target digit on the new side. Each trial can thus be thought of as containing two task periods, each lasting for 3.25 s, separated by a 0.75-s gap for the potential shift. (The RSVP streams continued during this potential shift period, but the target number 5 was inhibited.) The task was programmed so that the onset of the first task period was always synced with the scanner pulse (time 0 for each trial). The attended locations of the two periods could either be the same (Hold-L and Hold-R conditions) or different (shift-LR and shift-RL conditions), as shown in Figure 2. The four trial types were randomly intermixed in each Eyes-fixed run so that participants could not predict the conditions before each trial.

The RSVP streams were composed of digits; the digit 5 was reserved as the target; other digits were presented randomly in a trial. In the RSVP, for every frame of 250 ms, there was a 1/3 chance that the target 5 would appear on the screen in one of middle (target) streams (when it appeared, it was randomly assigned to one of the target streams, and 5 never appeared in the flanker streams). The target presentation was temporally restricted so that two targets could not appear sequentially within 1 s, no matter whether it appeared in the cued or uncued stream.

Stimuli in the Eyes-move task were similar, except that instead of fixating at the screen center, the fixation cross could appear at one of two potential fixation locations at the start of each trial, 2.5° to the left and right of the screen center, and there were three columns of RSVP streams, located at the far left, center, and far right of the screen, each centered 2.5° away from the nearest fixation location (Fig. 1*B*). On half of the trials, the fixation cross remained in the same position for the entire trial (no-saccade trials); on the other half of trials, the fixation cross jumped to the other fixation location halfway through the trial (saccade trials). Subjects were instructed to fixate their eyes on the fixation cross and saccade to the new location whenever it moved.

Each Eyes-move run was subdivided into four miniblocks (eight trials each). Two of these blocks contained the spatiotopic reference frame condition, where subjects were instructed to attend to the central RSVP stream regardless of where their eyes were. This condition was cued at the beginning of the miniblock as “attend screen center,” and the letter cue “C” appeared above and below the current fixation to remind subjects of the attended location. The other two miniblocks contained the retinotopic reference frame conditions, where subjects were instructed to attend to an RSVP stream defined relative to fixation, i.e., “left of the cross” or “right of the cross.” These conditions were cued as such at the beginning of the miniblock, and with letters L and R, respectively, during the trial. The order of these four miniblocks was randomized in each run. Participants always knew which reference frame condition they were in, but they could not predict either the initial fixation location or whether they would have to make a saccade or not on each trial.

Each trial in the Eyes-move task also lasted 8 s. As in the Eyes-fixed task, the scanner pulse was always synced with the onset of the first task period (time 0), the rest of the trial was designed so that the time course data would be as comparable as possible between Eyes-fixed and Eyes-move tasks. The initial fixation and letter cue alone appeared 1 s before the start of the trial (onset of the RSVP streams). The first task period lasted 3 s and the second 2.5 s, separated by a 1-s gap for a potential saccade. (The RSVP streams continued during this potential saccade period, but the target number 5 was inhibited.) There were another 0.5 s of blank gap after the second task period before the next trial began.

A summary of all conditions in the Eyes-move task is listed in Figure 2. The conditions were coded based on reference frame, attended location, and fixation location or saccade direction. For example, in spatiotopic blocks, no-saccade trials were coded as SpaC-Rfix (spatiotopic reference frame, attend center stream, fixation on the right cross) or SpaC-Lfix, and saccade trials were coded as SpaC-RLsac (spatiotopic reference frame, attend center stream, saccade from right to left cross) or SpaC-LRsac. In retinotopic blocks, no-saccade trials were coded as RetL-Rfix (retinotopic reference frame, attend stream left of fixation, fixation on the right cross), RetL-Lfix, RetR-Rfix, or RetR-Lfix; however, although our design included both left and right fixation location trials, we aggregated them into RetL-fix and RetR-fix to simplify our analyses. This is because the aggregated conditions did not involve a visual field difference, and any effect coming from pure fixation location difference is beyond the main scope of this study. Retinotopic saccade trials were coded as RetL-RLsac (retinotopic reference frame, attend stream left of fixation, saccade from right to left cross), RetL-LRsac, RetR-RL-sac, or RetR-LRsac. These conditions are all illustrated in Figure 2. In sum, our main MVPA analyses included a total of 10 task conditions (we also conducted a descriptive univariate analysis with different numbers of conditions; for details, see Results).

In both Eyes-fixed runs and Eyes-move runs, trial onset times were jittered, with intertrial intervals (ITIs) of 0, 2, and 4 s (50%, 35%, and 15% of trials, respectively), in a fast-event related fashion. An additional miniblock (16 s) of blank baseline was put in the beginning, middle and end of each run, respectively, where participants were instructed to keep fixated at the fixation cross. Participants completed four runs of Eyes-fixed task and 8 runs of Eyes-move task. In addition, they also completed two to four runs of the standard retinotopic mapping task (see details below, ROI section).

All stimuli were generated with the Psychtoolbox (Brainard, 1997) in MATLAB (MathWorks). Stimuli were displayed with a three-chips DLP projector onto a screen in the rear of the scanner (resolution 1280 × 1024 at 60 Hz). Participants viewed from a distance of 74 cm via a mirror above attached to the head coil.

### Eye tracking

Eye positions were recorded throughout the experiment when the calibration was reliable, using an MRI-compatible Eyelink remote eye-tracker at 500 Hz. Eye position data were used to ensure the participants kept their eyes on the fixation point and made eye movements following the fixation change. When eye position data were not available, the experimenters observed the participant’s eye through the camera and made sure that the participants were making eye movements as intended.

### fMRI acquisition

This study was done at the OSU Center for Cognitive and Behavioral Brain Imaging with a Siemens Prisma 3T MRI scanner using a 32-channel phase array receiver head coil. Functional data were acquired using a T2-weighted gradient-echo sequence (TR = 2000 ms, TE = 28 ms, flip angle 71°). The slice coverage was oriented ∼45° away from the AC-PC plane and placed to prioritize full coverage of occipital and parietal lobes, and then maximize coverage of temporal and frontal lobes (33 slices, 2 × 2 × 2 mm voxel, 10% gap). We also collected a high-resolution MPRAGE anatomic scan at 1-mm^3^ resolution for each participant. Each participant was scanned in one 2-h session.

### fMRI preprocessing

The fMRI data were preprocessed with Brain Voyager QX (Brain Innovation). All functional data were corrected for slice acquisition time and head motion and temporally filtered. Runs with abrupt motion >1 mm were discarded from later analyses, and the motion correction parameters were logged and input as nuisance variables into the general linear model (GLM). Spatial smoothing of 4-mm full-width at half-maximum (FWHM) was performed on the preprocessed data for univariate analyses, but not for multivoxel pattern analysis (MVPA). Data of each participant were normalized into Talairach space (Talairach and Tournoux, 1988). We used FreeSurfer to segment the white matter/gray matter boundaries from each participant’s anatomic scan, and imported the images into BrainVoyager for flattening. We extracted each participant’s cortical surface for each hemisphere in Talairach space, and inflated and flattened them into cortical surface space for retinotopic mapping. Other analyses were performed on volume space only.

### Regions of Interest (ROIs)

Our analyses focused on two a priori ROIs. These ROIs were our theoretical ROIs designed to look at attentional representations: bilateral area V4 (considered strongly modulated by attention; McAdams and Maunsell, 2000) and a functionally defined attention shift network (Yantis et al., 2002).

**Figure 3.**
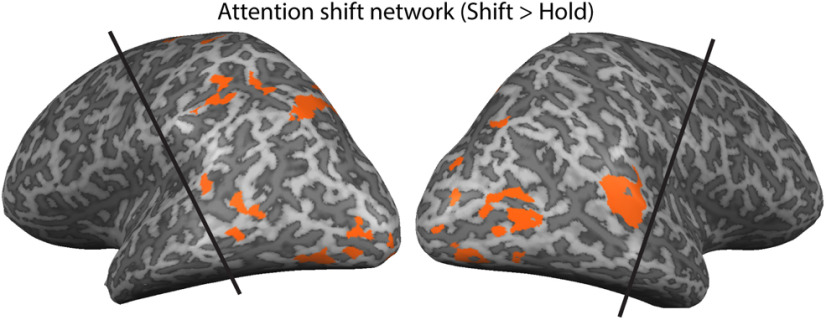
Functionally defined attention shift network, as described in the text. The volume maps were projected onto an inflated brain only for visualization purpose. The black lines demonstrate the approximate coverage (slightly different for each subject).

The attention shift network was functionally defined based on the group-level shift > hold univariate attention contrast in the Eyes-fixed task. For this contrast, we used a whole-brain multi-subject GLM in the Eyes-fixed task with five regressors (blank baseline plus the four Eyes-fixed conditions) and six nuisance regressors from the motion correction processing, with a canonical hemodynamic response function, to calculate β weights of each condition for each voxel. We then projected the contrasts of shift conditions versus hold conditions onto volume maps. All volume maps were corrected for cluster threshold at α = 0.05 level, using the BrainVoyager plugin “Cluster-level Statistical Threshold Estimator,” after which all significant voxel clusters were picked as the corresponding functional network. The attention shift network is shown in Figure 3 and Table 1. The attention shift network includes inferior parietal lobule (IPL) and temporal gyri, consistent with areas previously found in the literature (Corbetta et al., 1998; Beauchamp et al., 2001; Yantis et al., 2002; de Haan et al., 2008). Because of limited frontal coverage in our scanning protocol, our data only captured more posterior regions.

**Table 1:** Description of clusters in the attention shift network, including Talairach coordinates of the peak voxel, number of voxels, and *t* values

Areas	Hemisphere	TAL coordinates of peak voxel				
		*x*	*y*	*z*	# of voxels	*t* value (df = 11)
Superior Temporal Gyrus	R	63	−39	14	398	6.5813
	L	−53	−53	12	180	7.4536
Middle Temporal Gyrus	R	41	−61	6	389	6.5224
Inferior Occipital Gyrus	R	31	−81	−4	254	6.8871
	L	−37	−77	−4	226	5.7041
Inferior Parietal Lobule	L	−37	−37	42	1388	5.5973
Lingual Gyrus (posterior)	L	−11	−87	−14	163	5.2757
Lingual Gyrus (anterior)	L	−27	−61	4	133	4.6952
Superior Frontal Gyrus	L	−17	−13	74	146	4.9916

We used a standard phase-encoded retinotopic mapping localizer (Sereno et al., 1995) to define visual area V4 for each participant. In the retinotopic mapping scans, a rotating wedge with high-contrast radial checkerboard patterns was presented on the screen and flickered at 4 Hz. The 60° wedge stimulus covered eccentricity from 1.6° to 16° and was rotated either clockwise or counter-clockwise for seven cycles with a period of 24 s per cycle. Participants were instructed to fixate at the center fixation of the screen, and press the button every time when the fixation dot changed color from dark gray to light gray. A pair of clockwise and counterclockwise runs were combined in the analyses. One or two pairs of runs (i.e., two to four runs) were obtained for each participant. After preprocessing, the brain data were analyzed in custom MATLAB code and projected onto the flattened brains as surface maps in Brain Voyager. Bilateral V4 boundaries were defined based on these surface maps. We then used the task > baseline contrast from the Eyes-fixed runs to further constrain the retinotopic ROIs to regions visually activated by this task.

In addition to these a priori ROIs, we also defined a *post hoc* network for exploratory analyses, the “retinotopic-hold” network, based on the cross-task similarity searchlight results (see details below), corrected for cluster threshold in the same way as above. ROI results for this *post hoc* network are presented for descriptive purposes only, as the datasets used to define and analyze were not fully independent.

Finally, in the Extended Data (Tables 2-1, 3-1), we also report results from two additional, comparison ROIs to capture generic visual activation (bilateral area V1) and deactivation (functionally-defined task negative network). Area V1 was defined using the same retinotopic mapping procedure as V4, and the task-negative network was defined based on the group-level baseline > task contrast in the Eyes-fixed task, where task included all four Eyes-fixed task conditions.

### Multivoxel Pattern Analyses (MVPAs)

For all MVPA analyses below, we imported corresponding GLM data to MATLAB with BrainVoyager’s BVQXtools MATLAB toolbox, and all subsequent analyses were done using custom MATLAB code.

#### Within-task MVPA (split-half correlation-based analyses)

We first performed MVPA within the Eyes-Fixed and Eyes-move tasks (e.g., comparing the Eyes-fixed conditions to each other), using the split-half correlation-based method (Haxby et al., 2001) for each participant and each ROI/network. This split-half procedure is necessary for the within-task analysis to avoid confounds driven by the diagonal cells in the correlation matrix. (The split-half procedure is not necessary for cross-run analyses; see Cross-task pattern similarity analysis in later section.) We obtained GLMs for odd runs and even runs separately for each participant; each GLM had five regressors for the Eyes-fixed task (blank baseline plus the four Eyes-fixed conditions) and 11 regressors for the Eyes-move task (blank baseline plus the 10 Eyes-move conditions from Fig. 2), as well as six nuisance regressors from the motion correction processing. For the following analyses, we focused on non-baseline conditions. For each GLM, we normalized the voxel data (β weights) by subtracting the mean response across all non-baseline conditions from the response of each individual condition, for each voxel. This standard demeaning procedure (Coutanche, 2013) was done within each fold of split-half data. The response patterns (voxel-wise β weights after de-meaning) for each condition in the even runs were then correlated with the patterns for each condition in the odd runs, generating a correlation matrix for each task. The correlation coefficients were transformed into z-scores using Fisher’s r-to-z transform.

We then calculated the following types of information based on the correlation matrix. In the Eyes-fixed task: information about shift execution (holding vs shifting attention), hold attention location (holding left vs holding right), and shift direction (shifting leftward vs shifting rightward). In the Eyes-move task: information about saccade execution (saccade vs no saccade), saccade direction (saccade leftward vs saccade rightward), and reference frame (retinotopic attention task vs spatiotopic attention task). Specifically, we picked out cells in the matrix that reflected the same type of information (“within-category” correlations, e.g., holding attention correlated with holding attention), and cells that reflected the different type of information (“between-category” correlations, e.g., holding attention correlated with shifting attention). The information index was then calculated by subtracting the mean correlation values of “different” cells from those of “same” cells. A significantly-positive information index value would indicate that there is some amount of information of this type in the ROI.

#### MVPTC analyses

The first step of analyses described above used regular whole-trial GLMs, which modeled the whole 8 s (four TR) trial as a single event. However, since trials contained a potential attention shift or saccade halfway through, the initial analysis might fail to capture some dynamic brain representations. Thus, we also performed time course analyses using finite impulse response (FIR) GLM analyses with 10 time points, on the same conditions as above. Time point 0 (TP0) corresponds to the start of the first task period in each trial (i.e., the onset of RSVP stimuli). We fed those FIR GLMs into MVPAs (i.e., MVPTC, modified from Chiu et al., 2012). Taking each time point as a separate dataset, we performed similar analyses as above to calculate the information indices. The result figures show all 10 TPs in the FIR, but our statistical analyses focus on three TPs that capture critical time periods in the trial, accounting for BOLD signal lag: TP3 (before the shift/saccade happened), TP4 (capturing the shift/saccade), TP5 (after the shift/saccade). It is also important to clarify that at the behavioral time period corresponding to BOLD signals at TP3, participants did not know yet whether there would be an attentional shift or not (in Eyes-fixed task), or a saccade or not (in Eyes-move task), because the trials were intermixed; however, it was predictable that if there would be a shift/saccade, what direction the shift/saccade would be, based on the attention location or the eye location within the first half of a trial.

#### Cross-task pattern similarity analysis

To directly compare the similarity between the brain activity patterns of covert attention during Eyes-fixed and Eyes-move tasks, we performed a cross-task pattern similarity analysis for both whole-trial and time course β weights. Because the Eyes-fixed and Eyes-move tasks were performed in separate runs, we used GLMs of all runs instead of split-half to increase power; that is, we took Eyes-fixed runs and Eyes-move runs as the two datasets for the correlation analysis. After de-meaning the voxel-wise responses in the same way as above, we calculated the z-scored correlation matrix comparing each condition in the Eyes-fixed task to each saccade condition in the Eyes-move task. We then calculated the pattern similarity between the following four pairings by averaging the z-scored correlation coefficients of corresponding cells in the matrix: retinotopic-to-hold, retinotopic-to-shift, spatiotopic-to-hold, spatiotopic-to-shift. The similarity data were submitted to a 2 (Eyes-move conditions: retinotopic and spatiotopic) by 2 (similarity to Eyes-fixed conditions: similarity-to-hold and similarity-to-shift) ANOVA. In this ANOVA analysis, a main effect of similarity to Eyes-fixed conditions would indicate that both retinotopic and spatiotopic attention (across saccades) are represented more similarly to hold (or shift) attention than shift (or hold), an interaction would indicate relatively greater similarity between retinotopic and holding attention and between spatiotopic and shifting attention (or relatively greater similarity between spatiotopic and holding attention and between retinotopic and shifting attention). To help illustrate the result, we also plot the difference in pattern similarity between spatiotopic-to-shift minus spatiotopic-to-hold correlations, and the difference between retinotopic-to-shift minus retinotopic-to-hold correlations; the difference between these difference scores reflects the interaction term from the ANOVA analysis above.

#### Whole-brain searchlight on cross-task pattern similarity analysis

Finally, we performed MVPA searchlight analyses (Kriegeskorte et al., 2006) to search across the entire slice coverage, for clusters that might show patterns of interest outside our a priori ROIs. The approach is similar to what is described above; instead of taking a priori ROIs, we searched through individual brains iteratively with a “moving” ROI, defined as a sphere of radius three voxels. On each iteration, MVPTC analyses were performed as described above on each ROI sphere, and z-scored correlation values were assigned to the center voxel of this ROI sphere to form z-maps for each subject. We generated such searchlight maps for three measures: the difference in similarity between spatiotopic-to-shift and spatiotopic-to-hold correlations, the difference between retinotopic-to-shift and retinotopic-to-hold correlations, and their interaction (i.e., the interaction term in the ANOVA described in the prior section). Specifically, we focused only on TP4, which theoretically captured the time point at shift/saccade. To generate these difference maps, we first generated four searchlight maps for each individual subject, indexing each pair of correlations: retinotopic-to-hold, spatiotopic-to-hold, retinotopic-to-shift, and spatiotopic-to-shift. We calculated the difference maps by comparing (subtracting) the appropriate similarity maps for each subject accordingly. The resulting searchlight difference and interaction maps for each individual were then spatially smoothed with a 4-mm FWHM kernel to facilitate group analyses. Group *t* value maps were constructed using two-tailed *t* tests comparing the values for each voxel against zero, correcting for cluster threshold in the same way as above. For the first two difference maps, a positive *t* value for a given voxel indicates that spatiotopic/retinotopic attention is represented more similar to shifting than holding attention. For the final interaction map, a positive *t* value for a given voxel indicates that retinotopic attention across saccades is represented more similar to holding attention at fixation, and spatiotopic more similar to shifting attention (i.e., the “retinotopic-hold/spatiotopic-shift” pattern), a negative *t* value indicates that retinotopic attention across saccades is represented more similar to shifting attention at fixation, and spatiotopic more similar to holding attention (i.e., the “spatiotopic-hold/retinotopic-shift” pattern).

## Results

Our main theoretical question of interest is whether maintaining retinotopic or spatiotopic attention across saccades is represented relatively more like holds (or shifts) of attention at fixation. Our primary focus is thus on the cross-task similarity results from our a priori attention-related ROIs (along with an exploratory searchlight analysis). Before presenting these cross-task MVPA results, we first report the behavioral, univariate, and within-task MVPA results to establish the sensitivity of the paradigm and provide context for the cross-task results.

### Initial results 1: behavior

To evaluate participants’ behavioral performance, we defined hits as correctly pressing a button within 1 s in response to a 5 target at the attended location, and false alarms as incorrectly pressing a button when there was no 5 target within 1 s at the attended location. We calculated the hit rate by dividing the total number of hits in each trial by the total number of targets at the attended location (trials with 0 targets were omitted). We also calculated the false alarm rate by dividing the total number of false alarms in each trial by the total number of frames when there was no target presented in the attended RSVP stream. D-prime was calculated by subtracting z-scored false alarm rates from z-scored hit rates.

Because of a coding mistake for data logging, two subjects did not have reliable behavioral responses logged and were excluded from the analyses of behavioral performance. For the remaining 10 subjects, the mean hit rate in Eyes-fixed task was 66.17% (±5.07% SD), and the mean false alarm rate was 0.52% (±0.14% SD); in Eyes-move task, the mean hit rate was 65.67% (±5.70%) and the mean false alarm rate was 0.50% (±0.18%). These two tasks were designed to be hard to make sure that participants maintained attention on the cued location, so it is reasonable that participants’ performance was not at ceiling. The d-prime measurements in both tasks were well above zero, *t*s ≥ 15.239, *p*s ≤ 0.001, Cohen’s *d*s ≥ 4.819, and there was no significant difference between the two tasks, *t*_(9)_ = 0.217, *p *=* *0.833, Cohen’s *d *=* *0.069. In addition, there were no significant differences of d-prime between hold and shift attention in Eyes-fixed task, between saccade and no saccade trials in Eyes-move task, and between spatiotopic and retinotopic attention, all *t*s ≤ 2.083, *p*s ≥ 0.067, Cohen’s *d*s ≤ 0.659.

### Initial results 2: univariate comparisons

**Figure 4.**
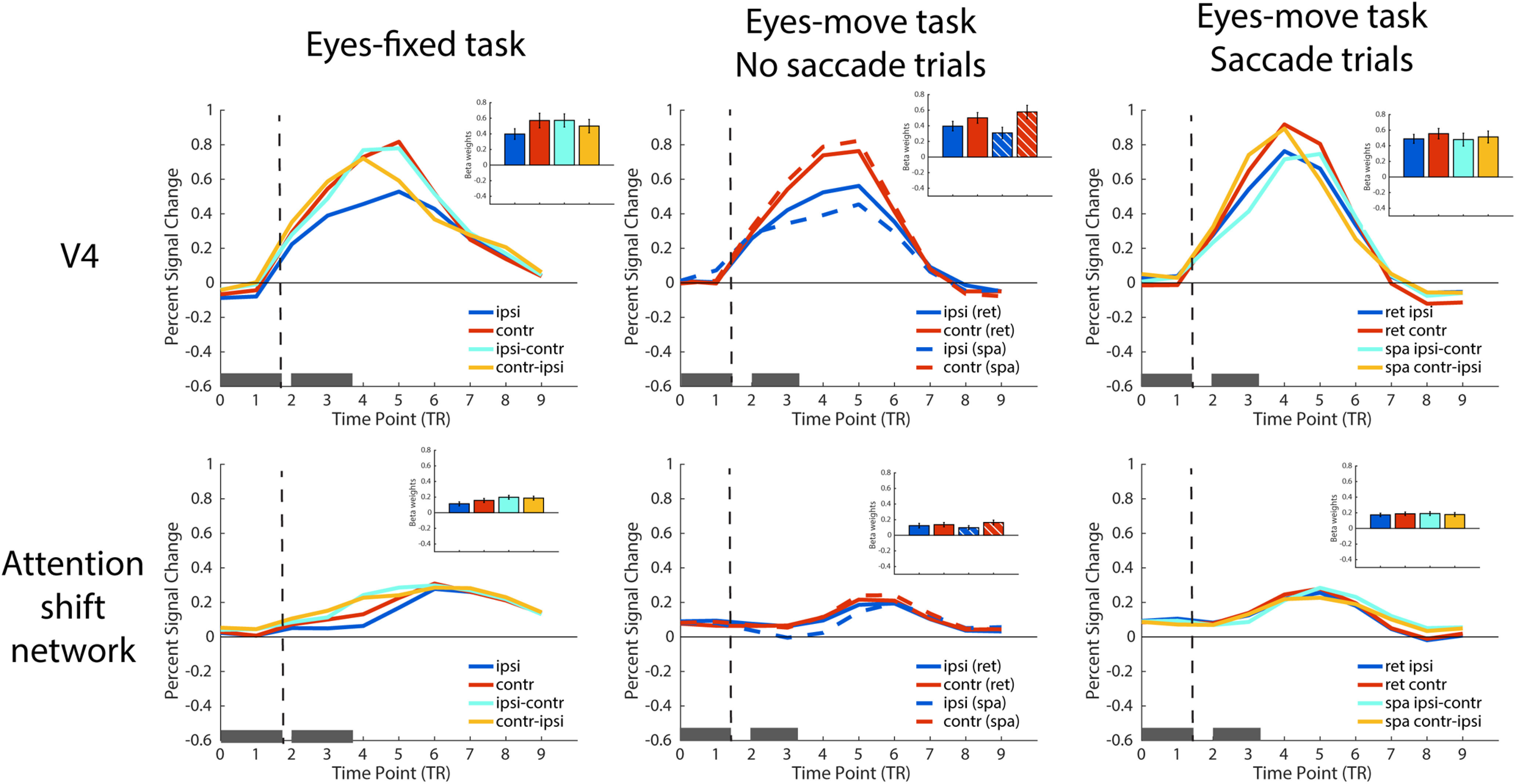
Univariate results of Eyes-fixed task (left column), Eyes-move task with no saccade trials (middle column), and Eyes-move task with saccade trials (right column). The pair of gray boxes along the *x*-axis in each plot indicates the time duration of the two task periods in the trial, and the vertical dashed lines indicate the onset of shift or saccade cues. Inset bar plots show the whole-trial β weights for each condition in each ROI/network, color-coded in the same way as the corresponding FIR timecourse plots. Error bars represent standard errors of the mean (SEM).

To give a general view of how the brain activity looks like for each condition, Figure 4 plots the percent signal change in the time course as well as the univariate β weights for our two a priori attention-related ROIs. To better illustrate, we recoded the conditions to plot them according to whether the attended side was ipsilateral/contralateral relative to the ROIs in each hemisphere, and further collapsed across the RL and LR saccade directions in retinotopic saccade trials (that is, only 8 conditions were shown in Eyes-move results). To make it comparable for each condition, we subtracted the percent signal change or β weights of fixation baseline from all other conditions, in both Eyes-fixed and Eyes-move task. As shown in Figure 4, there was a separation in the attention shift network between holding and shifting attention around TP4, as well as a clear pattern of contralateral attentional modulation in V4.

### Initial results 3: MVPA of shifting versus holding attention (Eyes-fixed)

**Figure 5.**
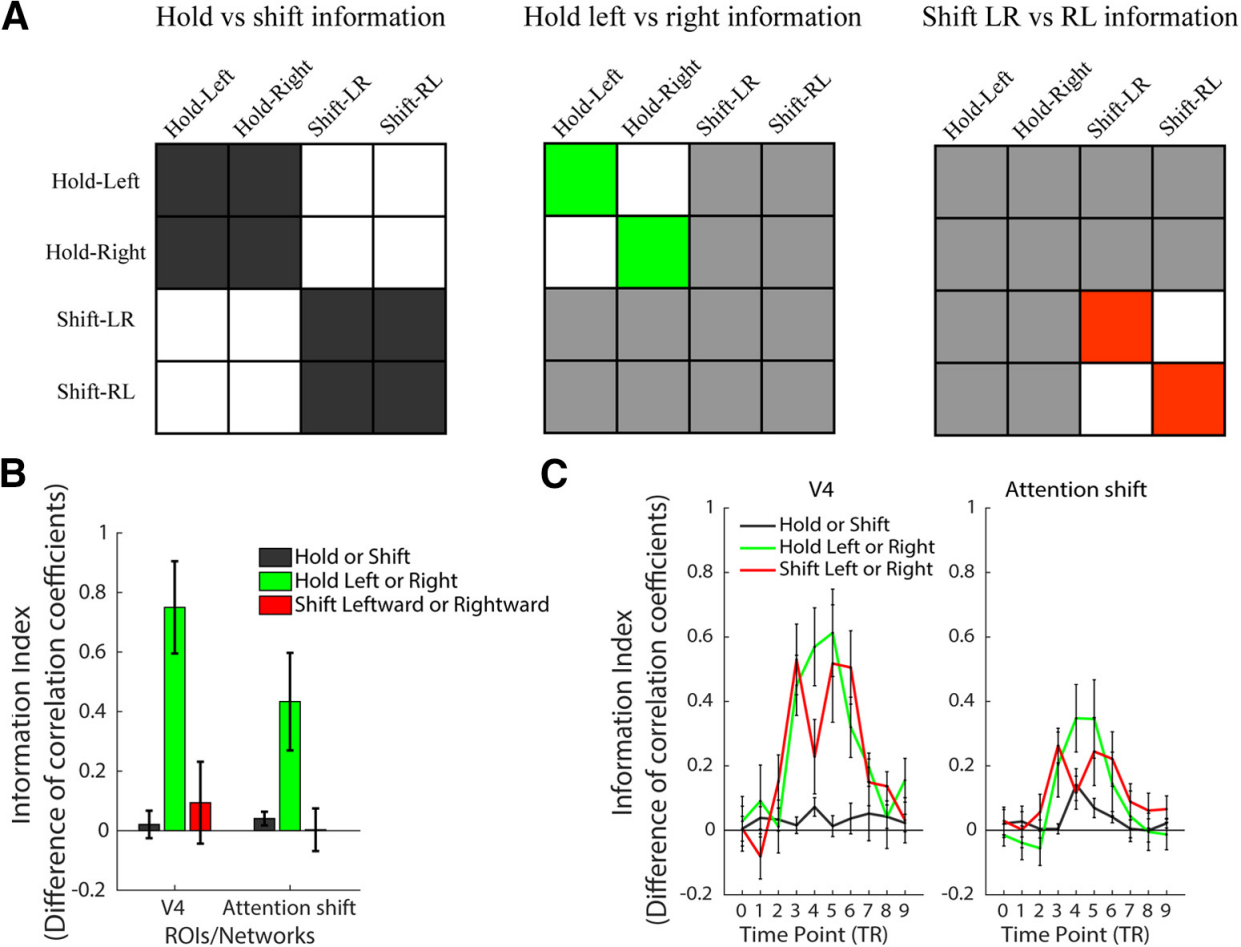
MVPA and MVPTC analyses and results of Eyes-fixed tasks. ***A***, Hypothetical matrices for hold versus shift, hold left versus right, and shift LR versus RL information. Cells colored in dark gray, green, and red are the within-category correlations, and white cells are the between-category correlations. Light gray cells are not used in the corresponding analysis. The information index is calculated by subtracting the z-scored between-category correlation coefficients from the z-scored within-category correlation coefficients. ***B***, The information index of each type of information in each ROI/network. ***C***, The information index timecourse of each type of information at 10 time points, in each ROI/network. Error bars represent SEM.

For the Eyes-fixed task, we examined whether we could decode from the brain patterns information about shift execution (holding vs shifting attention trials), about hold attention location (attending left vs right stream on hold trials), and about shift direction (shift left-right vs shift right-left trials; Fig. 5*A*). From each of our a priori ROIs/networks, we conducted correlation-based MVPA on the whole-trial GLM β weights (Fig. 5*B*). We also examined how these three types of information develop over the time course of the trials (MVPTC), by using β weights from the FIR GLMs (Fig. 5*C*). Table 2 lists *t* test statistics for each of these comparisons for the whole-trial βs and critical time points TP3, TP4, and TP5, corresponding to the critical behavioral time periods before the shift/saccade happened, around the shift/saccade, and after the shift/saccade was done.

**Table 2:** Statistical tests of information indices in each ROI/network, separately for whole-trial analyses and time points of interest in the time course analyses

	V4	Attention shift network
Hold or shift	*t*_(11)_ = 0.451, *p *=* *0.661, *d *=* *0.130 TP3: *t*_(11)_ = 0.608, *p *=* *0.555, *d *=* *0.176 TP4: *t*_(11)_ = 2.507, *p *=* *0.029, *d *=* *0.724* TP5: *t*_(11)_ = 0.394, *p *=* *0.701, *d *=* *0.114	*t*_(11)_ = 1.755, *p *=* *0.107, *d *=* *0.507 TP3: *t*_(11)_ = 0.278, *p *=* *0.787, *d *=* *0.080 TP4: *t*_(11)_ = 2.853, *p *=* *0.016, *d *=* *0.823** TP5: *t*_(11)_ = 2.316, *p *=* *0.041, *d *=* *0.668*
Hold L or hold R	*t*_(11)_ = 4.843, *p *<* *0.001, *d *=* *1.380** TP3: *t*_(11)_ = 4.818, *p *<* *0.001, *d *=* *1.391** TP4: *t*_(11)_ = 4.709, *p *<* *0.001, *d *=* *1.359** TP5: *t*_(11)_ = 4.521, *p *<* *0.001, *d *=* *1.305**	*t*_(11)_ = 2.645, *p *=* *0.023, *d *=* *0.764** TP3: *t*_(11)_ = 2.025, *p *=* *0.068, *d *=* *0.585 TP4: *t*_(11)_ = 3.326, *p *=* *0.007, *d *=* *0.960** TP5: *t*_(11)_ = 2.834, *p *=* *0.016, *d *=* *0.818^**^
Shift leftward or rightward	*t*_(11)_ = 0.682, *p *=* *0.510, *d *=* *0.197 TP3: *t*_(11)_ = 4.840, *p *<* *0.001, *d *=* *1.397** TP4: *t*_(11)_ = 1.975, *p *=* *0.074, *d *=* *0.570 TP5: *t*_(11)_ = 2.839, *p *=* *0.016, *d *=* *0.820**	*t*_(11)_ = 0.040, *p *=* *0.969, *d *=* *0.012 TP3: *t*_(11)_ = 4.903, *p *<* *0.001, *d *=* *1.415** TP4: *t*_(11)_ = 2.273, *p *=* *0.044, *d *=* *0.656** TP5: *t*_(11)_ = 2.310, *p *=* *0.041, *d *=* *0.667^*^
Saccade or no saccade	*t*_(11)_ = 1.452, *p *=* *0.175, *d *=* *0.419 TP3: *t*_(11)_ = 2.056, *p *=* *0.064, *d *=* *0.594 TP4: *t*_(11)_ = 3.305, *p *=* *0.007, *d *=* *0.954** TP5: *t*_(11)_ = 2.014, *p *=* *0.069, *d *=* *0.581	*t*_(11)_ = 4.432, *p *=* *0.001, *d *=* *1.279** TP3: *t*_(11)_ = 2.598, *p *=* *0.025, *d *=* *0.750** TP4: *t*_(11)_ = 2.956, *p *=* *0.013, *d *=* *0.853** TP5: *t*_(11)_ = 7.249, *p *<* *0.001, *d *=* *2.093**
Saccade leftward or rightward	*t*_(11)_ = 2.730, *p *=* *0.020, *d *=* *0.788** TP3: *t*_(11)_ = 4.113, *p *=* *0.002, *d *=* *1.187** TP4: *t*_(11)_ = 7.401, *p *<* *0.001, *d *=* *2.136** TP5: *t*_(11)_ = 3.370, *p *=* *0.006, *d *=* *0.973**	*t*_(11)_ = 1.771, *p *=* *0.104, *d *=* *0.511 TP3: *t*_(11)_ = 4.420, *p *=* *0.001, *d *=* *1.276** TP4: *t*_(11)_ = 1.775, *p *=* *0.104, *d *=* *0.512 TP5: *t*_(11)_ = 3.615, *p *=* *0.004, *d *=* *1.044**
Retinotopic or spatiotopic	*t*_(11)_ = 0.504, *p *=* *0.625, *d *=* *0.145 TP3: *t*_(11)_ = 0.067, *p *=* *0.948, *d *=* *0.019 TP4: *t*_(11)_ = 0.101, *p *=* *0.921, *d *=* *0.029 TP5: *t*_(11)_ = 0.819, *p *=* *0.430, *d *=* *0.237	*t*_(11)_ = 0.074, *p *=* *0.943, *d *=* *0.021 TP3: *t*_(11)_ = 0.816, *p *=* *0.432, *d *=* *0.236 TP4: *t*_(11)_ = 1.295, *p *=* *0.222, *d *=* *0.374 TP5: *t*_(11)_ = 1.095, *p *=* *0.297, *d *=* *0.316

*N* = 12.

* statistical significance at *p *<* *0.05.

** statistical significance at *p *<* *0.05 (Holm–Bonferroni corrected for multiple *post hoc* comparisons, separately across ROIs/networks for whole-trial MVPA, and across three TPs for MVPTC).

10.1523/ENEURO.0186-20.2021.t2-1 Extended Data Table 2-1 Statistical tests of information indices in V1 and task negative network, separately for whole-trial analyses and time points of interest in the time-course analyses. N=12. Download Table 2-1, DOCX file.

For the information about shift execution (holding vs shifting attention trials), we did not find significant information with the whole trial MVPA analyses in the attention shift network nor in V4. However, recall that trials were 8 s long, and the hold and shift trials were designed to be identical for the majority of the trial, except the transient shift occurring midway through the trial. Indeed, when analyzing the time course in the attention shift network, we did find significant information about shift execution at the critical TP4. There was a weak effect at TP5 that did not pass correction for multiple comparisons, and no significant information about shift execution for TP3, before the shift happened. The MVPTC analyses thus successfully captured a transient change in activity pattern around the time when the shifts happened, in the attention shift network (in V4 we found information about shift execution at TP4 that did not pass correction for multiple comparisons). [Note that the attention shift network was defined by the univariate contrast of shift > hold (with the whole-trial βs), so these MVPA results are not completely independent, although a univariate effect alone (linear transform) could not drive a correlation-based MVPA difference; nonetheless, these MVPTC results are useful as a validity check, and the remaining analyses that we focus on below are fully independent of the ROI definitions.]

For the information about which location was attended on hold trials (holding left vs right stream), we found significant information in the whole-trial MVPA, in both the attention shift network and V4. MVPTC showed that this information was sustained for the duration of the trial and was significant at TP3, TP4, and TP5 in the attention shift network and V4, with the only exception at TP3 in the attention shift network. This is consistent with the behavioral task on these hold trials, in that participants maintained attention in one location throughout the entire trial.

The analogous analysis for the shift attention trials examines information about covert attention shift direction (shift left-right vs shift right-left trials). We did not find significant information in either ROI with whole-trial β weights. The time course analyses may give some insight into why. Interestingly, the MVPTC took a different shape than for the previous analyses; here, instead of peaking at the critical TP4, the information was actually greater at TP3 and TP5 than at TP4 in both ROIs/networks. In V4, the shift direction information was significant at TP3 and TP5 but not TP4. This bimodal pattern also existed in the attention shift network numerically, but all three TPs were significant. It should be noted that in our design, the direction of the shifting was perfectly confounded with the location participants attended to before and after the shift. Thus, the bimodal pattern may reflect a dynamic representation of which location was being attended in the first half of the trial (peaking at TP3), and then after the attention shift in the second half of the trial (peaking at TP5), rather than reflecting information about the shift direction itself.

### Initial results 4: MVPA of attention maintained across saccades (Eyes-move)

**Figure 6.**
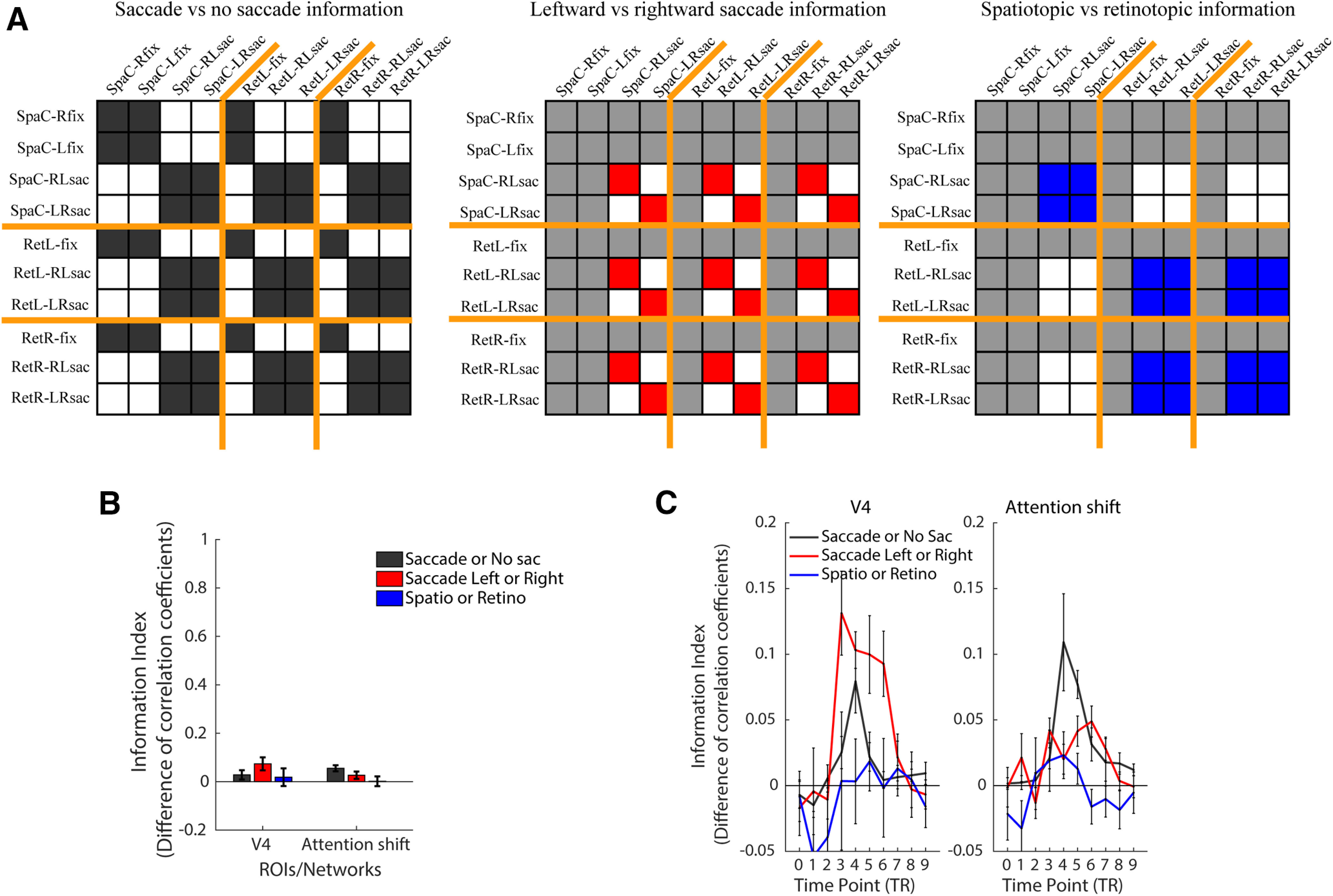
MVPA and MVPTC analyses and results of the Eyes-move task. ***A***, Hypothetical matrices for information about: saccade versus no saccade, leftward versus rightward saccade, and spatiotopic versus retinotopic attention (across saccades). Orange lines separate conditions in spatiotopic (“attend center”), retinotopic left (“attend left of cross”), and retinotopic right (“attend right of cross”) blocks. Cells colored in dark gray, red, and blue are the within-category correlations, and white cells are the between-category correlations. Light gray cells are not used in the corresponding analysis. The information index is calculated by subtracting the z-scored between-category correlation coefficients from the z-scored within-category correlation coefficients. ***B***, The information index of each type of information in each ROI/network; the scale is the same as Figure 5*B*. ***C***, The information index of each type of information at 10 time points, in each ROI/network; the scale is different from Figure 5*B*,*C* and panel ***B***. Error bars represent SEM. Extended analyses are shown in Extended Data Figures 6-1, 6-2, 6-3.

For the Eyes-move task, we used a similar approach of whole-trial MVPA followed by MVPTC to examine information about saccade execution (saccade vs no-saccade trials), and on saccade trials, about the saccade direction (leftward vs rightward saccade) and reference frame (retinotopic vs spatiotopic attention; Fig. 6; statistics in Table 2).

10.1523/ENEURO.0186-20.2021.f6-1 Extended Data Figure 6-1 Extended data showing MVPTC results of information about the hemifield attended (left or right) before and after the saccade separately (in the Eyes-move task). The index values of each type of information at 10 time points are plotted for each ROI/network. Error bars represent SEM. Results show that we could decode which hemifield was being covertly attended both before and after the saccade. Download Figure 6-1, TIF file.

10.1523/ENEURO.0186-20.2021.f6-2 Extended Data Figure 6-2 Extended data showing information of retinotopic versus spatiotopic attention in searchlight analyses, for time points 3, 4, and 5 separately. This whole-brain analysis is analogous to Figure 6, blue condition (information about spatiotopic vs retinotopic). Red areas show significant information after cluster-threshold correction at *p* < 0.05. The viewing angle for each row is left lateral, left medial, right lateral, and right medial, respectively. Download Figure 6-2, TIF file.

10.1523/ENEURO.0186-20.2021.f6-3 Extended Data Figure 6-3 Extended data showing univariate differences, based on whole-trial βs, between saccade and no saccade conditions in the Eyes-move task (top), and between retinotopic and spatiotopic conditions in the Eyes-move task (bottom). For each contrast, significant clusters in the positive direction are shown in green and negative in orange. Maps were cluster threshold corrected at *p* < 0.05. For spatiotopic > retinotopic contrast, the only significant cluster found was located in the left hemisphere, so only the left lateral viewing angle is shown here. Download Figure 6-3, TIF file.

For the information about saccade execution (saccade vs no-saccade trials), we found significant information in whole-trial MVPA analyses in the attention shift network. When looking at time course analyses, we found that the information was represented significantly in V4 and the attention shift network at TP4, corresponding to the behavioral time period of saccade execution. In the attention shift network, this information was also significant at both TP3 and TP5. *Post hoc t* tests comparing the information indices at TP3/TP5 to TP4 showed that the information at the critical TP4 was significantly greater than at TP3, *t*_(11)_ = 2.772, *p *=* *0.018, Cohen’s *d *=* *0.800, but information at TP4 was only numerically larger than at TP5, *t*_(11)_ = 0.946, *p *=* *0.364, Cohen’s *d *=* *0.273. It is possible that saccade preparation and saccade execution might have elongated the process and thus blurred the effect temporally in the attention shift network.

For the information about saccade direction (right-left saccade vs left-right saccade), we found weak information that did not pass correction with whole-trial MVPA in V4, but not in the attention shift network. In the MVPTC, the saccade direction information was significant in all three time points in V4, and at TP3 and TP5 in the attention shift network. Some of the time courses appeared to have a similar bimodal shape for information about saccade direction as above for covert attention shift direction, perhaps again driven by information about attended hemisphere over time (Extended Data Fig. 6-1). Interestingly, although both V4 and the attention shift network represented information on saccade execution and saccade direction information, V4 seems to have more information about saccade direction, whereas the attention shift network had more information about saccade execution.

Finally, we did not find reference frame information (retinotopic-attention vs spatiotopic-attention trials) in whole-trial MVPA analyses in either ROI. In the time course analysis, no time points were significant in V4 or the attention shift network. Thus, our attentionally-modulated ROIs did not appear to directly differentiate which reference frame participants were maintaining attention in, although as noted above, they contained information about which location was being attended at any given time, and whether saccades were being executed.

### Main results: cross-task similarity analysis of covert attention at fixation and across saccades

The above results demonstrate that brain regions sensitive to attentional modulation (V4 and the attentional shift network) represent information about covert attention shifts and about saccade execution. Now the key question is, how do representations of covert attention during fixation compare to covert attention maintained across saccades? Depending on the reference frame, both spatiotopic and retinotopic attention could be thought of as “hold” or “shift” attention tasks: spatiotopic attention is maintained in the same location relative to the screen, but shifted relative to our eyes, whereas retinotopic attention is the opposite. Is one or both of these tasks represented more similarly to holding attention in some brain regions, and/or more similarly to shifting attention elsewhere in the brain?

**Figure 7.**
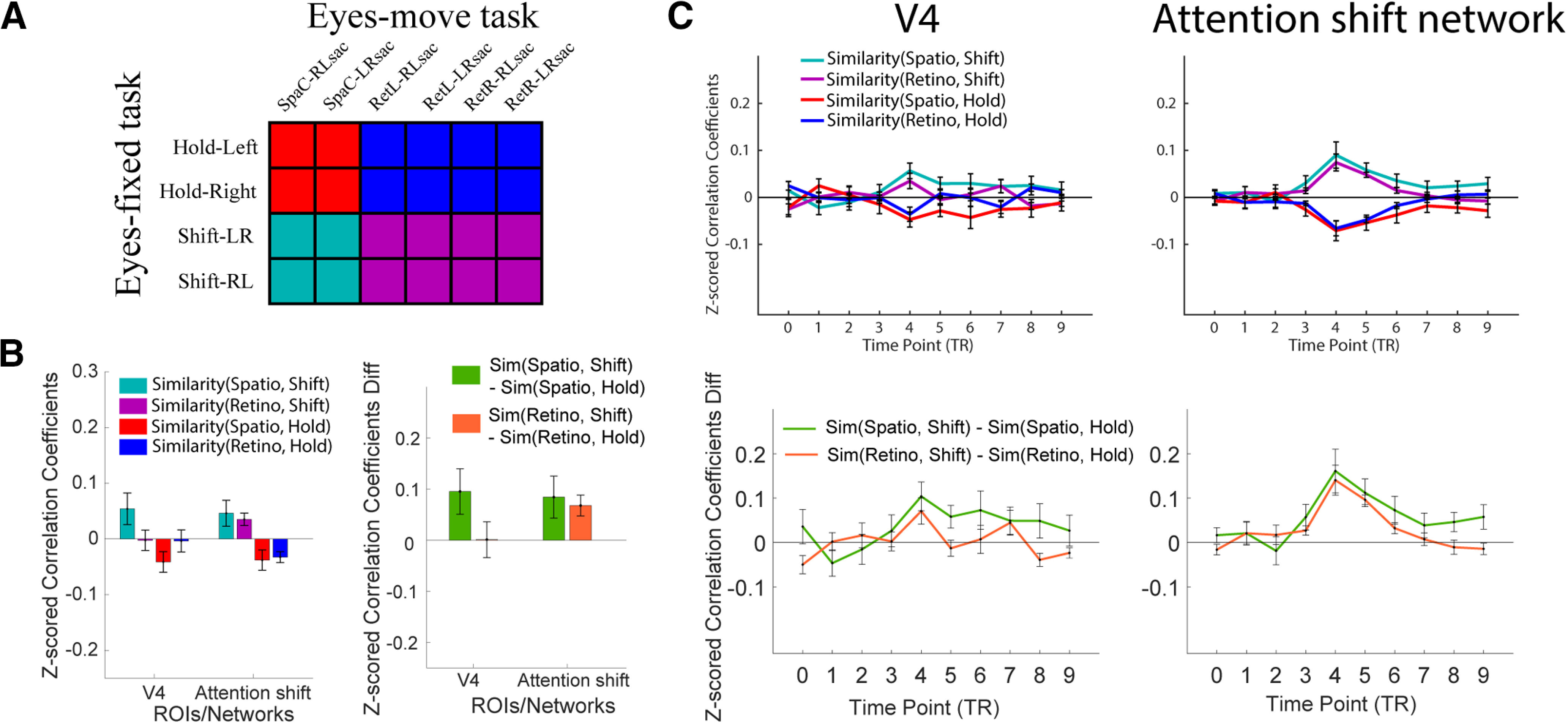
Cross-task similarity analyses in a priori ROIs/networks. ***A***, A hypothetical matrix indicating each combination of similarity: retinotopic-to-hold (blue), retinotopic-to-shift (magenta), spatiotopic-to-hold (red), and spatiotopic-to-shift (cyan). ***B***, ***C***, Pattern similarity (z-scored correlation coefficients) for each combination of conditions, for each ROI/network. ***B***, Pattern similarity based on whole-trial β weights. Left, Similarity for each of the four cross-task pairings. Right, Pattern similarity difference scores, showing [spatio-to-shift minus spatio-to-hold] and [retino-to-shift minus retino-to-hold]. ***C***, Pattern similarity time courses based on FIR β weights for each of 10 time points. Top row, For each of the four cross-task pairings. Bottom two, Pattern similarity difference scores as in ***B***. Error bars represent SEM. Note that the roughly symmetrical patterns of the time course plots are likely because of the de-meaning step of subtracting the grand mean activity across conditions for each time point’s MVPA analysis, but it does not influence the interpretation for the main effects and interactions (see Materials and Methods).

To answer these questions, we analyzed the pattern similarity between Eyes-fixed conditions and Eyes-move conditions (Fig. 7*A*). Rather than calculate information indices, in this cross-task MVPA analysis we directly compare the representational similarity scores for each cross-task pair of conditions (i.e., similarity between retinotopic and hold, between spatiotopic and hold, between retinotopic and shift, and between spatiotopic and shift). We also plot the difference scores between spatiotopic-to-shift minus spatiotopic-to-hold correlations, and retinotopic-to-shift minus retinotopic-to-hold correlations. The results of this analysis are shown in Figure 7*B*,*C*, and statistics from the 2 × 2 ANOVA are reported in Table 3 for each ROI/network at each critical time point, as well as for the whole trial data.

**Table 3:** Statistics of 2 × 2 repeated-measure ANOVAs for each ROI at TP3, TP4, and TP5 respectively, on pattern similarity between Eyes-fixed conditions (hold and shift attention) and Eyes-move conditions (spatiotopic and retinotopic attention), separately for whole-trial analyses and time points of interest

	V4	Attention shift network
Main effect of similarity to Eyes-fixed conditions (similarity-to-hold; similarity-to-shift)	*F *=* *8.367, *p *=* *0.015, η_p_^2^ = 0.432** TP3: *F *=* *2.549, *p *=* *0.139, η_p_^2^ = 0.188 TP4: *F *=* *13.113, *p *=* *0.004, η_p_^2^ = 0.544** TP5: *F *=* *4.269, *p *=* *0.063, η_p_^2^ = 0.280	*F *=* *18.892, *p *=* *0.001, η_p_^2^ = 0.632* TP3: *F *=* *15.604, *p *=* *0.002, η_p_^2^ = 0.587** TP4: *F *=* *15.293, *p *=* *0.002, η_p_^2^ = 0.582** TP5: *F *=* *29.311, *p *<* *0.001, η_p_^2^ = 0.727**
Main effect of Eyes-move conditions (spatiotopic; retinotopic)	*F *=* *0.486, *p *=* *0.500, η_p_^2^ = 0.042 TP3: *F *=* *0.510, *p *=* *0.490, η_p_^2^ = 0.044 TP4: *F *=* *0.922, *p *=* *0.358, η_p_^2^ = 0.077 TP5: *F *=* *0.184, *p *=* *0.676, η_p_^2^ = 0.016	*F *=* *0.291, *p *=* *0.601, η_p_^2^ = 0.026 TP3: *F *=* *0.291, *p *=* *0.600, η_p_^2^ = 0.026 TP4: *F *=* *5.514, *p *=* *0.039, η_p_^2^ = 0.334* TP5: *F *=* *0.827, *p *=* *0.383, η_p_^2^ = 0.070
Interaction	*F *=* *1.672, *p *=* *0.223, η_p_^2^ = 0.132 TP3: *F *=* *0.182, *p *=* *0.678, η_p_^2^ = 0.016 TP4: *F *=* *0.727, *p *=* *0.412, η_p_^2^ = 0.062 TP5: *F *=* *3.407, *p *=* *0.092, η_p_^2^ = 0.236	*F *=* *0.091, *p *=* *0.768, η_p_^2^ = 0.008 TP3: *F *=* *0.640, *p *=* *0.441, η_p_^2^ = 0.055 TP4: *F *=* *0.348, *p *=* *0.567, η_p_^2^ = 0.031 TP5: *F *=* *0.351, *p *=* *0.566, η_p_^2^ = 0.031

* statistical significance at *p *<* *0.05.

** statistical significance at *p *<* *0.05 (Holm–Bonferroni corrected for multiple *post hoc* comparisons, separately across ROIs/networks for whole-trial β weights, and across three TPs for time course β weights).

10.1523/ENEURO.0186-20.2021.t3-1 Extended Data Table 3-1 Statistics of 2×2 repeated-measure ANOVAs for V1 and task negative network at TP3, TP4, and TP5 respectively, on pattern similarity between Eyes-fixed conditions (hold & shift attention) and Eyes-move conditions (spatiotopic & retinotopic attention), separately for whole-trial analyses and time points of interest. Download Table 3-1, DOCX file.

In the whole-trial MVPA analysis, there was a significant main effect of similarity-to-shift versus similarity-to-hold in both V4 and the attention shift network, in that the representational similarity scores were generally higher when correlating the Eyes-move conditions with the Eyes-fixed shift attention condition, compared with with the Eyes-fixed hold attention condition. In the MVPTC analysis, this main effect was significant at critical time point TP4 in both ROIs, and also at the neighboring time points TP3 and TP5 in the attention shift network.

*Post hoc* analyses with whole-trial data reveal that in V4, maintaining spatiotopic attention across saccades was represented marginally more similarly to shift than to hold (*t*_(11)_ = 2.141, *p *=* *0.056, Cohen’s *d *=* *0.618), and there was no significant difference between retinotopic-to-shift and retinotopic-to-hold correlations in the whole-trial analysis (*t*_(11)_ = 0.032, *p *=* *0.975, Cohen’s *d *=* *0.009). In the attention shift network, spatiotopic was marginally more similar to shift than to hold (*t*_(11)_ = 2.056, *p *=* *0.064, Cohen’s *d *=* *0.594), and retinotopic was significantly more similar to shift than to hold (*t*_(11)_ = 3.306, *p *=* *0.007, Cohen’s *d *=* *0.954). The interaction in the ANOVA was not significant in either ROI in the whole trial analysis. In the MVPTC, *post hoc* analyses at the critical time point TP4 showed that both retinotopic and spatiotopic attention across saccades were significantly more similar to shift than to hold in both V4 and the attention shift network (*t*s ≥ 2.387, *p*s ≤ 0.036, Cohen’s *d*s ≥ 0.689). The similarity-to-shift effect also seemed to be numerically greater for the spatiotopic compared with retinotopic attention condition in V4, but again this interaction was not significant, nor was it significant in the attention shift network.

### Exploratory results: whole-brain cross-task similarity searchlight

The above results suggest that both retinotopic and spatiotopic attention across saccades are represented more like shifts than holds of attention at fixation, with no significant interaction in our a priori attention ROIs indicating that one reference frame is represented more strongly than the other. As an exploratory analysis, we next asked: are there other areas in the brain that might show differential similarity patterns? We performed a searchlight analysis for a significant interaction effect at the critical time point TP4, as described in Materials and Methods.

**Figure 8:**
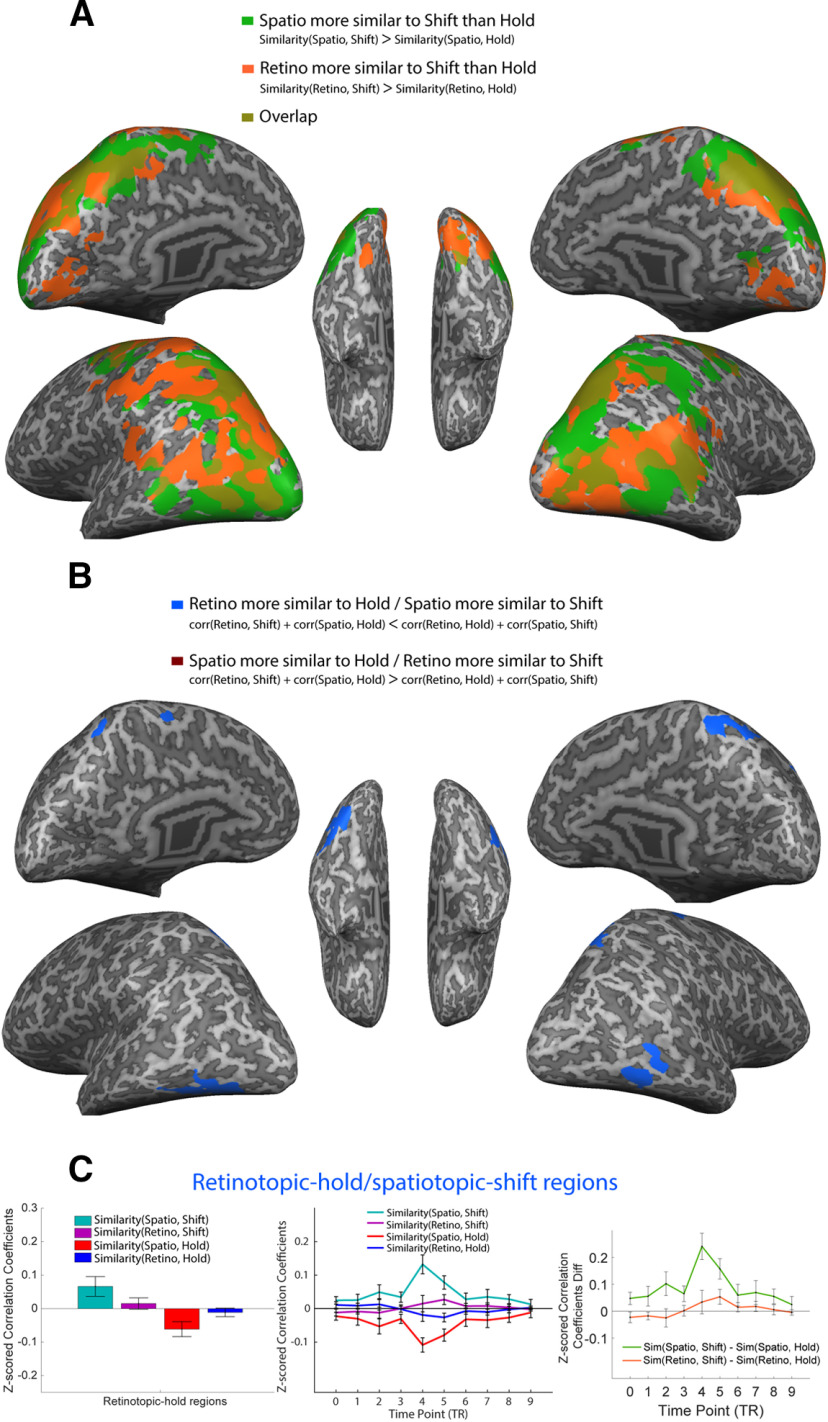
Cross-task pattern similarity, whole-brain searchlight analyses. ***A***, Regions showing significant difference between retinotopic-shift similarity and retinotopic-hold similarity (orange), and regions showing significant difference between spatiotopic-shift similarity and spatiotopic-hold similarity (green). Overlapping regions shown in brown. Note, no regions showing higher similarity to holding than shifting (for either comparison) survived the cluster threshold correction. ***B***, Regions showing a significant interaction effect. Regions exhibiting a significant retino-hold/spatio-shift pattern shown in blue; regions exhibiting a significant spatio-hold/retino-shift pattern shown in scarlet (no clusters passed significance threshold for this contrast). All searchlights are based on cross-task MVPTC, using the pattern correlation difference at TP4, with direction of contrast as indicated in the legends. The searchlight maps were corrected for cluster-threshold in the same way as other brain maps. Searchlight analyses were conducted on the volume maps and projected onto an inflated brain for visualization purpose. ***C***, Pattern similarity in the whole-trial (left) and in time courses (middle) for each combination of conditions, and the difference scores for similarity-to-shift and similarity-to-hold (right), shown for the retino-hold/spatio-shift areas extracted from ***B*** (all voxels averaged into single network; for separate plots for each individual area, see Extended Data Figure 8-1). Plots are for illustrative purposes only to explore the specific pattern driving the significant interaction. Error bars represent SEM.

In Figure 8*A*, we first show the difference score searchlight maps between spatiotopic-to-shift versus spatiotopic-to-hold, and retinotopic-to-shift versus retinotopic-to-hold. These difference score maps revealed that throughout the brain, both retinotopic and spatiotopic attention across saccades are widely represented as more similar to shifting attention than holding attention, consistent with our ROI findings.

Critically, the interaction map (Fig. 8*B*) allowed us to extract potential regions that significantly differentiate retinotopic and spatiotopic representations via one of two interaction patterns: (1) retinotopic relatively more similar to hold, and spatiotopic relatively more similar to shift; or (2) spatiotopic relatively more similar to hold, and retinotopic relatively more similar to shift. The searchlight revealed four clusters (Fig. 8*B*; Table 4), all with the retinotopic-hold/spatiotopic-shift pattern. The clusters were located in ventral areas and superior parietal regions bilaterally, which were in later visual hierarchy in both ventral and dorsal pathways. No regions with the spatiotopic-hold/retinotopic-shift pattern survived the cluster threshold correction.

**Table 4:** Description of clusters in regions with the retinotopic-hold pattern, including Talairach coordinates of the peak voxel, number of voxels, and *t* values

Areas	Hemisphere	TAL coordinates of peak voxel				
		*x*	*y*	*z*	# of voxels	*t* values (df = 11)
Parahippocampal gyrus	R	39	−49	4	691	4.0403
Fusiform gyrus	L	−37	−59	−12	708	4.9133
Precuneus	R	11	−60	67	884	3.9127
Paracentral Lobule	L	−3	−41	60	472	3.4624

For illustration purposes, we plot the cross-task similarities for the regions identified in the searchlight (Fig. 8C; plots for separate clusters in Extended Data Fig. 8-1). Note that this analysis is circular; we show the interaction patterns here for descriptive purposes only. The interaction in these regions seems to be primarily driven by the spatiotopic comparisons, particularly the high similarity between spatiotopic and shifting attention.

## Discussion

In summary, we found that both spatiotopic and retinotopic attention across saccades were represented more similarly to shifting compared with holding attention at fixation, especially in the attention shift network. Our a priori attention ROIs did not reveal a significant interaction between retinotopic and spatiotopic similarity, but our exploratory searchlight analysis revealed some brain regions where maintaining spatiotopic attention was represented more similarly to shifting attention and maintaining retinotopic attention was relatively more similar to holding attention (retinotopic-hold/spatiotopic-shift regions), with no brain regions displaying the opposite pattern.

In addition to these primary results, we were able to uncover several other signatures of covert attention during fixation and across saccades from the multivoxel activation patterns in various brain regions. First, pattern similarity results from within the Eyes-fixed task support the validity of our design and analyses. In the visual and attention shift areas, we could decode which location the participants were holding attention at, even dynamically in the time course, consistent with existing findings that attention modulates the activity in visual areas (Desimone and Duncan, 1995) and pattern activities in shift-related areas can be used to decode attention in the left versus right hemifield (Gmeindl et al., 2016). In the Eyes-move task, we could similarly decode which hemifield was being covertly attended both before and after the saccade (Extended Data Fig. 6-1). We could also reliably decode from the Eyes-fixed task whether a covert attention shift was executed in the middle of the trials, specifically at the critical time point TP4 which corresponds to the transient shift, consistent with time course decoding results about shift execution with SVM in Chiu et al., 2012. In the Eyes-move task, information about saccade execution emerged at TP4 in V4, and at all of TP3, TP4, and TP5 in the attention shift network. Below we discuss how our study contributes to the existing literature and informs our understanding of the mechanisms of covert attention across saccades.

### Representational patterns for covert attention across saccades

How spatial attention is maintained/updated in particular reference frames across saccades has been an open question in the literature, and it is actively debated with various paradigms whether one reference frame is more native or dominant, and thus requires less updating across saccades, than the other (Melcher and Morrone, 2003; Golomb et al., 2008; Crespi et al., 2011; Golomb and Kanwisher, 2012a,b; Satel et al., 2012; Turi and Burr, 2012; Zimmermann et al., 2013; Fabius et al., 2016; Fairhall et al., 2017; Shafer-Skelton and Golomb, 2018). In the case of spatial attention, it has been argued that attention pointers proactively remap to compensate for saccades and maintain spatiotopic attention (Cavanagh et al., 2010; Rolfs et al., 2011; Marino and Mazer, 2018), but also that attention might linger in retinotopic coordinates even after a saccade (Golomb et al., 2008, 2010; Jonikaitis et al., 2013; Golomb, 2019). More generally, spatiotopic remapping signals have been found in several brain regions, including monkeys’ lateral intraparietal area (LIP; Duhamel et al., 1992), superior colliculus (SC; Walker et al., 1995), frontal eye field (FEF; Umeno and Goldberg, 1997), and striate and extrastriate cortex (Nakamura and Colby, 2002), and human visual and parietal cortex (Merriam et al., 2003, 2007). Higher-level visual and parietal areas in particular have also been a focus of much debate over dominant reference frames for neuronal receptive fields (Duhamel et al., 1997; Snyder et al., 1998), fMRI adaptation (McKyton and Zohary, 2007; Zimmermann et al., 2016; Fairhall et al., 2017; Baltaretu et al., 2018), functional organization (d’Avossa et al., 2007; Ward et al., 2010; Crespi et al., 2011; Golomb and Kanwisher, 2012b), and attentional modulation (Golomb et al., 2010; Rawley and Constantinidis, 2011).

In the current study, we approached this question from a different angle. As introduced earlier, eye movements distinguish the two reference frames in a way that maintaining retinotopic attention can be considered as “holding” a location relative to the eyes and “shifting” relative to the world, and maintaining spatiotopic attention can be considered as shifting relative to the eyes and holding relative to the world. To our knowledge, the current study is the first attempt to directly compare the brain activity patterns of covert attention maintained/updated in the periphery across saccades and during fixation. We found that in the predefined attention shift network, maintaining both retinotopic and spatiotopic attention across saccades evoked more similar representational patterns to covertly shifting attention than to covertly holding attention at fixation. Perhaps this is not surprising, given that both retinotopic and spatiotopic trials involved an eye movement, which is expected to engage attentional shifts as discussed below. In that sense, it is less notable that both retinotopic and spatiotopic resembled shifts more than holds per se; but the lack of a relative difference in representational similarity is intriguing. If attention were represented more natively in one reference frame, we may have predicted the other condition to show relatively more similarity to the shift condition. Our exploratory searchlight analysis did reveal some regions where maintaining spatiotopic attention across saccades was relatively more similar to shifting attention and retinotopic relatively more to holding, but no regions with the opposite pattern.

### Why were both retinotopic and spatiotopic attention represented like covert attention shifts?

Why did saccade trials of both reference frames have greater representational similarity to the covert shift attention trials than hold attention trials? We suggest that the answer may be related to our within-task similarity analyses finding that information about both covert attention shifts (in Eyes-fixed runs) and saccade execution (in Eyes-move runs) could be decoded from our attention shift network. As mentioned in the introduction, overt and covert attention have been found to involve overlapping brain areas (Corbetta et al., 1998; Nobre et al., 2000; Perry and Zeki, 2000; Beauchamp et al., 2001; de Haan et al., 2008). Our study differed from these studies in that the paradigm used in these previous studies typically involved overt and covert attention shifts aiming at the same target. In our design, we tried to disentangle the saccade execution from the allocation of top-down task-directed attention, by using top-down covert retinotopic and spatiotopic tasks. There are several possible interpretations of this overlap between representations of saccades and covert attention shifts in our task that could account for why the saccade and no-saccade trials may have produced differentiable activation patterns in the attention shift network, and why in these areas, saccade trials of both reference frames may have had greater representational similarity to the covert shift-attention trials in the cross-task similarity analysis.

One reason could be that covert shifting of attention is directly involved in making a saccade; i.e., the execution of the saccade required a presaccadic shift of attention toward the saccade target, and this initial covert shift was what was driving the representational similarity to the covert shift-attention trials. It has been widely shown that shifts of covert attention precede saccade execution (Godijn and Pratt, 2002; Peterson et al., 2004), and presaccadic attention is considered critical for determining the saccade endpoints to execute accurate saccades and enhancing perceptual representations of the saccade target (Gersch et al., 2004; Zhao et al., 2012). Even when the task is designed as attending to peripheral locations other than the saccade target, there is evidence that attention is still presaccadically shifted to the saccade target (Kowler et al., 1995). In our experiment, the information about saccade versus no saccade in the attention shift network emerged fairly early (around TP3), which could be related to the preparation stage (presaccadic shift stage) before the saccade was executed, potentially providing indirect support for this account.

Another potential account is that the Eyes-move task involved a covert shift of attention not related to execution of the saccade per se, but because of perisaccadic updating or remapping of the peripheral focus of attention, on both retinotopic and spatiotopic saccade trials. Previous studies involving spatiotopic remapping have found anticipatory remapping signals in the lateral IPS in monkeys (Duhamel et al., 1992), which could overlap with our parietal attention shift regions in humans. As described earlier, maintaining retinotopic attention can be seen as shifting attention relative to the screen/world, and maintaining spatiotopic attention can be seen as shifting attention relative to the eyes. It is possible that both types of attention in our task involved some updating process across saccades that engaged an attentional shift signal in this brain region, which would be consistent with our cross-task correlation results that both spatiotopic and retinotopic attention were more similar to shifting compared with holding attention in the attention shift network.

A third possibility could be that our Eyes-move task may have triggered a more generic temporary disengaging/reengaging of top-down attention; i.e., a transient change or shift of attention on saccade trials that might have occurred independently of saccade planning, executing, or remapping processes. For example, although our task and instructions were designed to encourage continuous attention, we cannot rule out the possibility that participants may have approached the task as a serial attention task (attend the relevant stream, then disengage to execute saccade, then reengage again on the relevant stream), instead of attending continuously on the relevant stream. Or the abrupt onset of the saccade cue might have captured attention and caused an involuntary shift of attention away from the to-be-attended location. In cases like these examples, a transient shift in attention on saccade trials may have evoked representationally similar patterns of activity as the goal-directed shifts of covert attention on fixation trials, without being directly related to the saccade itself. We found that both maintaining retinotopic and spatiotopic attention are represented as more similar to shifting than holding attention widely in dorsal and ventral areas (Fig. 8*A*), possibly revealing this generic representation of dynamic change. However, it is unlikely that this scenario could have accounted for our full pattern of results, particularly the searchlight findings of the interaction.

### Why did not we see greater differences between retinotopic and spatiotopic representations?

In general, we found less of a difference between retinotopic and spatiotopic conditions than what might have been expected. In analyses directly comparing the two reference frames, we did not reveal any representational difference between retinotopic and spatiotopic conditions in the whole-trial MVPA in the attention shift network or other ROIs. In the MVPTC analyses, significant information about retinotopic versus spatiotopic attention was only found in V1 at TP4, but not in other predefined ROIs/networks or time points (Extended Data Table 2-1). We further probed for retinotopic versus spatiotopic differences with whole-brain MVPTC searchlight (Extended Data Fig. 6-2) and a whole-brain univariate contrast (Extended Data Fig. 6-3), but only small scattered regions were found outside of our a priori ROIs.

The behavioral performance confirms that participants were allocating attention properly; but why did not we find greater differences in retinotopic versus spatiotopic patterns in our attention-related ROIs? One important consideration is that our task was designed to equate visual input across these two conditions. Both conditions contained constant, dynamic stimulation (RSVP streams) in the same three locations; the only difference was which of the streams, depending on which reference frame, was attended at any moment in time. This design is in contrast to a design commonly used in prior studies probing other aspects of reference frames across saccades, where only one stimulus is presented at a time, and retinotopic and spatiotopic conditions differ in terms of both stimulus-driven visual input and attentional locus (d’Avossa et al., 2007; McKyton and Zohary, 2007; Gardner et al., 2008; Crespi et al., 2011; Pertzov et al., 2011; Rawley and Constantinidis, 2011; Zimmermann et al., 2016; Golomb and Kanwisher, 2012a; Fairhall et al., 2017; Baltaretu et al., 2018).

Moreover, our analysis was designed to look for representational signatures associated with attending in a retinotopic or spatiotopic reference frame across saccades (i.e., how shift-like or hold-like they were), not to ask whether we could decode which particular retinotopic or spatiotopic locations were being attended. Early visual areas are known to be retinotopically organized (Sereno et al., 1995; d’Avossa et al., 2007; Gardner et al., 2008; Crespi et al., 2011; Golomb and Kanwisher, 2012b; Merriam et al., 2013), and we would expect that at least in these areas, attending to a particular retinotopic location across a saccade would look more similar to holding covert attention at that same retinotopic location during fixation than to shifting attention to a different retinotopic location (i.e., the brain activity pattern of RetL-RLsac would be more similar to Hold-L compared with Shift-LR, for example). Indeed, we could decode which hemifield(s) were attended on saccade trials (Extended Data Fig. 6-1), but this was not the goal of our study. Instead, the primary goal of the current study was to ask more broadly, whether the neural processes associated with maintaining attention in retinotopic (or spatiotopic) coordinates across saccades evoked more similar representational patterns to holding compared with shifting covert attention (at fixation). Thus, our analysis included correlations of conditions with both hemifields (e.g., similarity between retinotopic and hold attention includes correlations between RetL (with both RL and LR saccades) versus Hold-L, RetL versus Hold-R, RetR versus Hold-L, and RetR versus Hold-R; same for other cross-task correlations; for a more detailed comparison between matching and not matching hemifields, see Extended Data Fig. 7-1). This likely explains why we did not find a retinotopic-hold/spatiotopic-shift effect with the cross-task similarity searchlight analysis in early visual areas.

10.1523/ENEURO.0186-20.2021.f7-1 Extended Data Figure 7-1 Extended data showing an alternative way to analyze cross-task similarity, matching the hemispheric locations of covert attention, e.g., only correlating RetL and HoldL (RetR and HoldR) to calculate similarity between retinotopic and hold. Compared with the analysis in the paper (panel ***A***), here, we perform this alternative analysis based on matching the first half of the trial (before shift/saccade; panel ***B***) and matching the second half of the trial (after shift/saccade; panel ***C***). In panel ***B***, we can see that in both ROIs, at time point 3 when BOLD signals correspond to the first half of trial, all four pairs of correlations are positive, because we have explicitly matched the retinotopic location of attention for all. In the second half of the trial, now the correlations between retinotopic and hold (blue) and between spatiotopic and shift (cyan) are greater than those between retinotopic and shift (magenta) and spatiotopic and hold (red). This looks like the retinotopic attention condition has more representational similarity to holding attention, and the spatiotopic is more similar to shift, as reflected in the difference score plots. Panel ***C*** can be interpreted in a similar way. But again, this interpretation would be biased because we explicitly defined the conditions in terms of their retinotopic locations; thus, it is an unsurprising result. (Note, however, that the difference score plots are not symmetrical around zero, especially for the attention shift network; if the ROIs coded attention in a purely retinotopic manner, we would expect the difference curves to be of equal magnitude in opposite directions. Thus, even this retinotopically-biased analysis still reveals a pattern consistent with our original conclusions: that both retinotopic and spatiotopic attention in saccade trials carry some similarity to shifting attention.) Download Figure 7-1, TIF file.

10.1523/ENEURO.0186-20.2021.f8-1 Extended Data Figure 8-1 Extended data showing univariate activation (the first three columns) and cross-task pattern similarities (the last column), separately for each cluster of the retinotopic-hold regions from the exploratory searchlight analyses. The univariate activation plots were comparable to Figure 4 and the pattern similarity plots to Figures 7*C*, 8*B*. Download Figure 8-1, TIF file.

Instead, our cross-task pattern similarity analysis was better suited to reflect potential connections between the representations of covert attention across saccades and during fixations, independent of potential confounds from visual stimulation and hemifield-based attentional effects. Thus, it is telling that our predefined ROIs, particularly the attention shift network, did not show a difference in representational similarity between the retinotopic and spatiotopic reference frames in the cross-task similarity analysis, such that both were more representationally similar to shifting attention; but the exploratory searchlight analysis revealed some potential regions where maintaining spatiotopic attention was relatively more shift-like than retinotopic attention but not vice versa. This asymmetry may reflect the idea that retinotopic attention is the more “native” coordinate system for spatial attention (Golomb et al., 2008) and suggest potential regions for differentiating retinotopic and spatiotopic attention across saccades, though it is interesting that neither this pattern nor the opposite pattern was found within the attention shift network itself.

### Interactions across brain regions

Our findings suggest that maintaining spatiotopic and retinotopic attention across saccades may involve different types of updating that might be represented with hold and shift signals combined across different sets of regions. Some regions might be involved in both reference frames in a similar way (e.g., the attention shift network), and some other regions might use shift signals to further differentiate these two updating processes. That these other areas include bilateral anterior ventral areas and superior parietal regions, located in later visual hierarchy in both ventral and dorsal pathways, may hold further clues for understanding this complex process.

Our results support a close link between the neural mechanisms associated with covert attention shifts during fixation and maintaining retinotopic/spatiotopic attention across saccades in V4 and the attention shift network. In comparing the relative amounts and types of information present in the attention shift network versus area V4 patterns, we found an intriguing parallel; the attention shift network had relatively more information about the execution of covert attention shifts and saccades, while V4 had more information about the location of covert attention and the direction of saccades. This pattern aligns with the general understanding that the attention shift network is more involved in the execution of shifting spatial attention, and V4 in the modulation of spatial attention (Yantis et al., 2002). Outside the domain of perisaccadic processing, previous literature has shown that the attention shift network is associated with broad, domain-independent brain activity for transient shifts of attention (Yantis et al., 2002; Shomstein and Yantis, 2004, 2006; Greenberg et al., 2010; Chica et al., 2013; Gmeindl et al., 2016). Our findings comparing covert attention shifts with attention updating across saccades further indicate that the brain activity patterns associated with covert attention shifts may be widely and reliably involved in various domains, contexts, and tasks.

In summary, coordination between different brain networks/regions may support more flexible updating of attention across saccades in different contexts, raising interesting follow-up questions regarding how and when this process might be achieved mechanistically, and how it is related to behavior, development, and clinical implications.

## Synthesis

Reviewing Editor: Nicholas J. Priebe, University of Texas at Austin Decisions are customarily a result of the Reviewing Editor and the peer reviewers coming together and discussing their recommendations until a consensus is reached. When revisions are invited, a fact-based synthesis statement explaining their decision and outlining what is needed to prepare a revision will be listed below. The following reviewer(s) agreed to reveal their identity: Yu-Chin Chiu.

One reviewer and the editor evaluated the revision of your manuscript "Representations of covert attention across saccades: comparing pattern similarity to shifting and holding attention during fixation." The authors have substantially improved the manuscript, and the experimental design is novel. There remain only minor issues to be addressed, listed below.

-----------

REVIEWER #1

ADVANCES THE FIELD

The study does advance the field by being the first study to characterize the neural mechanisms for holding covert attention across saccades.

COMMENTS

In this revised manuscript, the authors have done additional analyses to address my comments and have streamlined the result sections to answer their main theoretical question. I have only two minor comments.

1. The authors have now articulated a main hypothesis on p6 line 92-94. However, I find the statement a little strange. On a first read, I was unsure whether the authors hypothesized about the activation pattern in the attention shift network or about the similarity between two modes of attention (maintaining retinotopic/spatiotopic attention across saccades vs. holding attention at fixation). The authors might want to revise that sentence.

2. In this manuscript, "spatiotopic attention" is a short version for "maintaining spatiotopic attention across saccades." A reader should know that by following the paper carefully. However, I still recommend using the long description whenever possible and suitable.
