# Supplementary material for: Neural Representations of Covert Attention across Saccades: Comparing Pattern Similarity to Shifting and Holding Attention during Fixation
Source: eNeuro. 2021 Mar 5;8(2):ENEURO.0186-20.2021. doi: 10.1523/ENEURO.0186-20.2021 (PMC8026251; doi:10.1523/ENEURO.0186-20.2021)
Supplement: Extended Data Table 2-1 — Statistical tests of information indices in V1 and task negative network, separately for whole-trial analyses and time points of interest in the time-course analyses. N=12. Download Table 2-1, DOCX file. [file enu-eN-NWR-0186-20-s07.docx]

Table 2-1 Statistical tests of information indices in V1 and task negative network, separately for whole-trial analyses and time points of interest in the time-course analyses. N=12.

|  | V1 | Task negative network |
| --- | --- | --- |
| Hold  or  Shift | *t*(11)=−0.102, *p*=.920, *d*=−0.030  TP3: *t*(11)=−0.104, *p*=.919, *d*=−0.030  TP4: *t*(11)=0.236, *p*=.818, *d*=0.068  TP5: *t*(11)=0.127, *p*=.902, *d*=0.037 | *t*(11)=0.016, *p*=.987, *d*=0.005  TP3: *t*(11)=0.751, *p*=.469, *d*=0.217  TP4: *t*(11)=1.451, *p*=.175, *d*=0.419  TP5: *t*(11)=−0.339, *p*=.741, *d*=−0.098 |
| Hold L  or  Hold R | *t*(11)=2.821, *p*=.017, *d*=0.815**  TP3: *t*(11)=4.224, *p*=.001, *d*=1.219**  TP4: *t*(11)=2.880, *p*=.015, *d*=0.831**  TP5: *t*(11)=3.071, *p*=.011, *d*=0.887** | *t*(11)=−0.237, *p*=.817, *d*=−0.068  TP3: *t*(11)=−0.498, *p*=.628, *d*=−0.144  TP4: *t*(11)=0.813, *p*=.434, *d*=0.235  TP5: *t*(11)=0.287, *p*=.780, *d*=0.083 |
| Shift leftward  or  rightward | *t*(11)=0.808, *p*=.437, *d*=0.233  TP3: *t*(11)=3.013, *p*=.012, *d*=0.870**  TP4: *t*(11)=1.247, *p*=.238, *d*=0.360  TP5: *t*(11)=2.772, *p*=.018, *d*=0.800** | *t*(11)=−1.334, *p*=.209, *d*=−0.385  TP3: *t*(11)=1.059, *p*=.312, *d*=0.306  TP4: *t*(11)=1.153, *p*=.273, *d*=0.333  TP5: *t*(11)=0.886, *p*=.394, *d*=0.256 |
| Saccade  or  no saccade | *t*(11)=1.522, *p*=.156, *d*=0.439  TP3: *t*(11)=0.819, *p*=.430, *d*=0.236  TP4: *t*(11)=2.552, *p*=.027, *d*=0.737*  TP5: *t*(11)=0.977, *p*=.350, *d*=0.282 | *t*(11)=2.527, *p*=.028, *d*=0.730*  TP3: *t*(11)=2.517, *p*=.029, *d*=0.727**  TP4: *t*(11)=2.888, *p*=.015, *d*=0.834**  TP5: *t*(11)=4.115, *p*=.002, *d*=1.188** |
| Saccade leftward  or  rightward | *t*(11)=0.021, *p*=.984, *d*=0.006  TP3: *t*(11)=1.329, *p*=.211, *d*=0.384  TP4: *t*(11)=0.072, *p*=.944, *d*=0.021  TP5: *t*(11)=1.219, *p*=.248, *d*=0.352 | *t*(11)=2.602, *p*=.025 *d*=0.751*  TP3: *t*(11)=3.699, *p*=.004, *d*=1.068**  TP4: *t*(11)=3.228, *p*=.008, *d*=0.932**  TP5: *t*(11)=3.484, *p*=.005, *d*=1.006** |
| Retinotopic  or  spatiotopic | *t*(11)=1.225, *p*=.246, *d*=0.354  TP3: *t*(11)=0.825, *p*=.427, *d*=0.238  TP4: *t*(11)=3.498, *p*=.005, *d*=1.010**  TP5: *t*(11)=1.138, *p*=.279, *d*=0.329 | *t*(11)=0.268, *p*=.794, *d*=0.077  TP3: *t*(11)=0.662, *p*=.522, *d*=0.1911  TP4: *t*(11)=2.367, *p*=.037, *d*=0.683*  TP5: *t*(11)=0.779, *p*=.452, *d*=0.225 |

* indicate statistical significance at p<.05

** indicate statistical significance at p<.05 (Holm-Bonferroni corrected for multiple post hoc comparisons, separately across ROIs/networks for whole-trial MVPA, and across three TPs for MVPTC)
