# Supplementary figures and images for: Neural Representations of Covert Attention across Saccades: Comparing Pattern Similarity to Shifting and Holding Attention during Fixation
Source: eNeuro. 2021 Mar 5;8(2):ENEURO.0186-20.2021. doi: 10.1523/ENEURO.0186-20.2021 (PMC8026251; doi:10.1523/ENEURO.0186-20.2021)

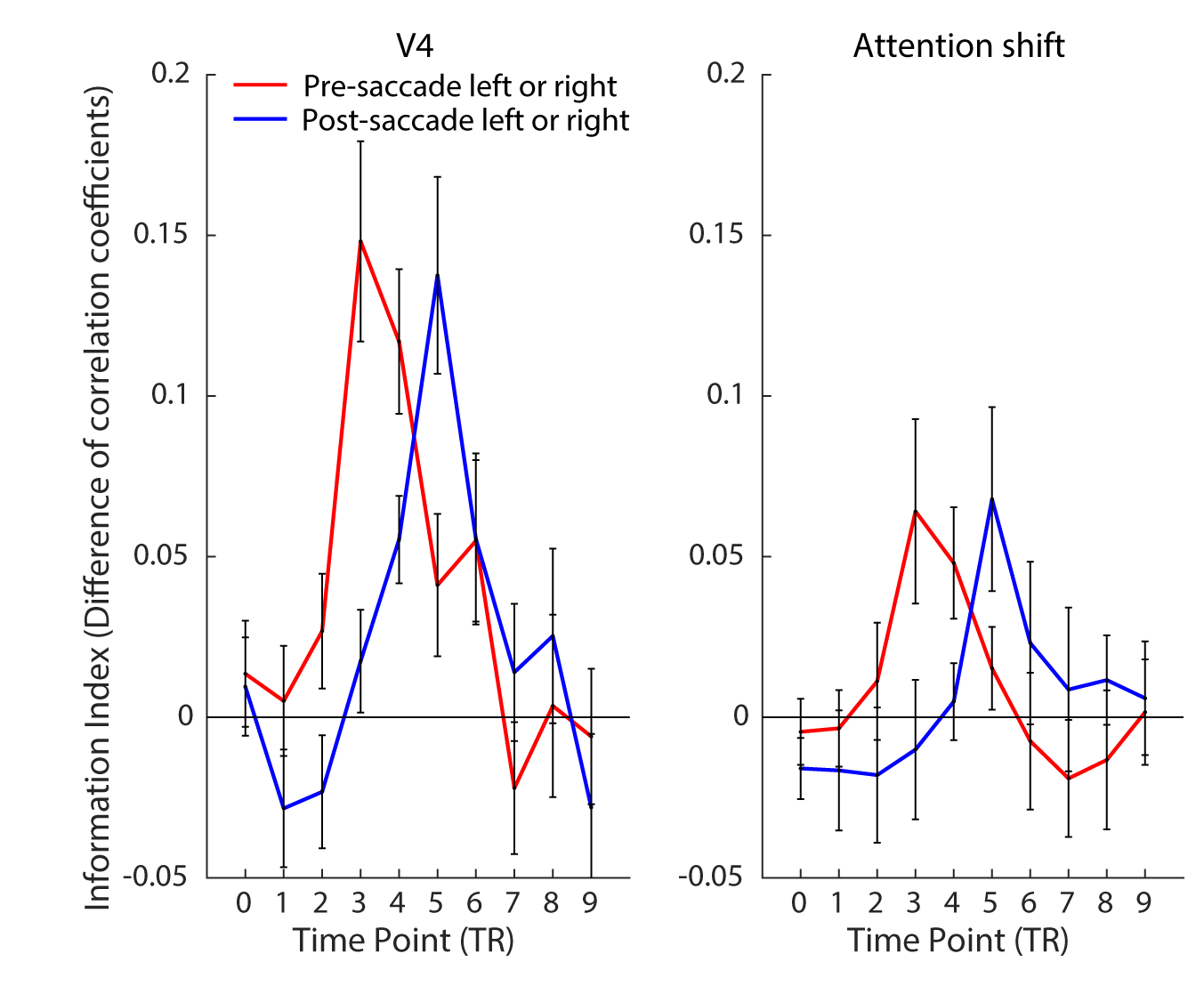

Supplement: Extended Data Figure 6-1 — Extended data showing MVPTC results of information about the hemifield attended (left or right) before and after the saccade separately (in the Eyes-move task). The index values of each type of information at 10 time points are plotted for each ROI/network. Error bars represent SEM. Results show that we could decode which hemifield was being covertly attended both before and after the saccade. Download Figure 6-1, TIF file. [file enu-eN-NWR-0186-20-s01.tif]

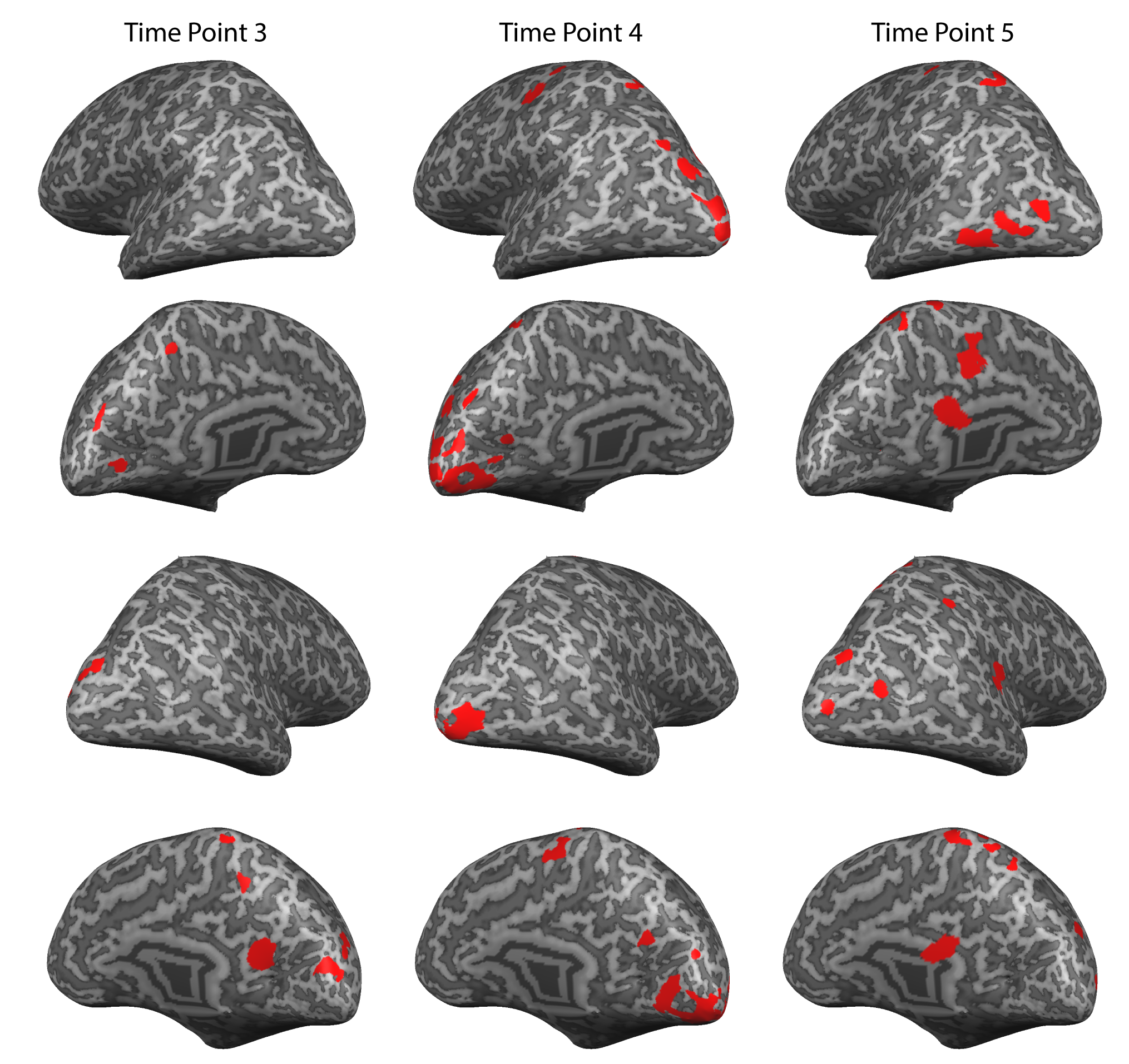

Supplement: Extended Data Figure 6-2 — Extended data showing information of retinotopic versus spatiotopic attention in searchlight analyses, for time points 3, 4, and 5 separately. This whole-brain analysis is analogous to Figure 6, blue condition (information about spatiotopic vs retinotopic). Red areas show significant information after cluster-threshold correction at p < 0.05. The viewing angle for each row is left lateral, left medial, right lateral, and right medial, respectively. Download Figure 6-2, TIF file. [file enu-eN-NWR-0186-20-s02.tif]

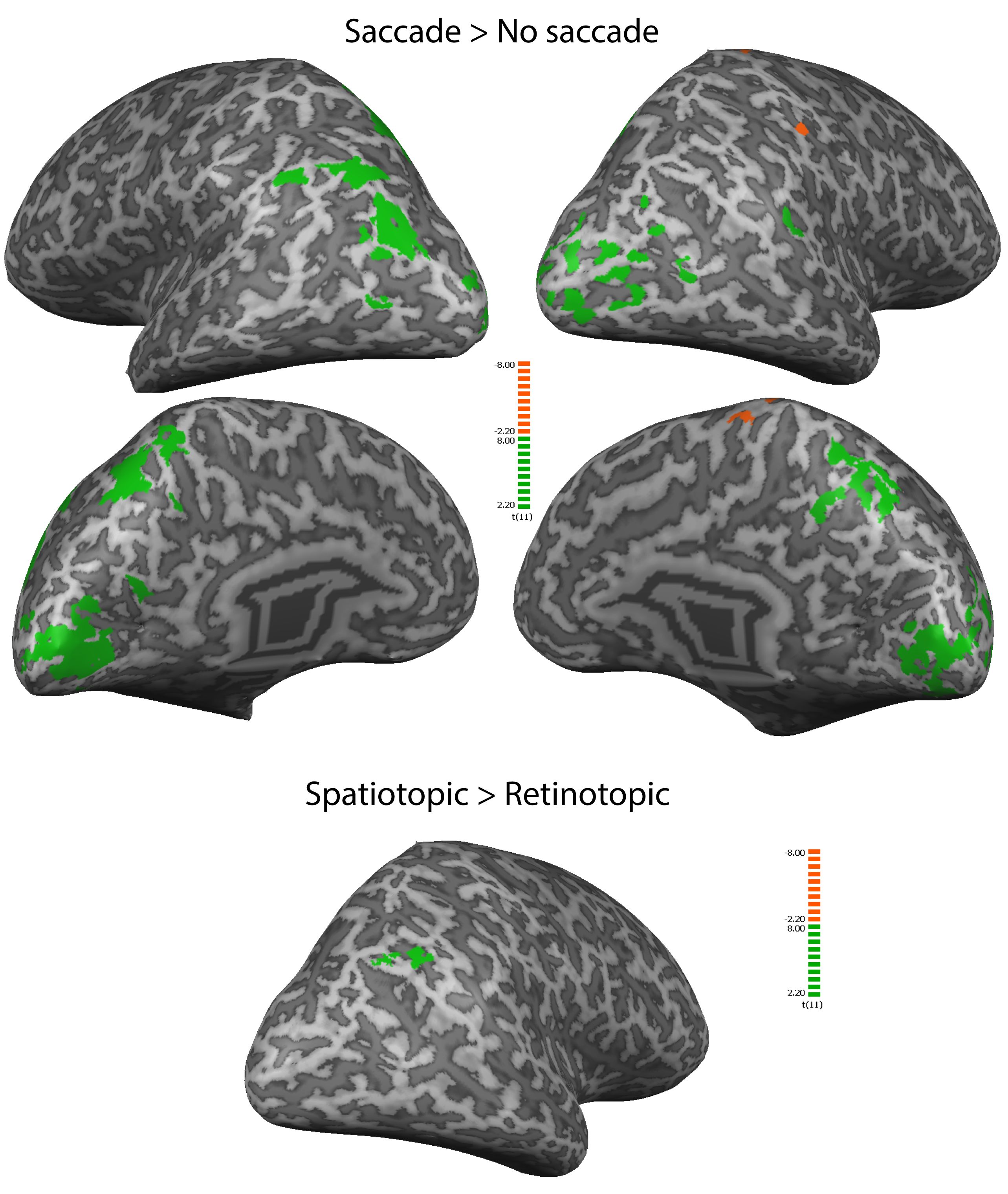

Supplement: Extended Data Figure 6-3 — Extended data showing univariate differences, based on whole-trial βs, between saccade and no saccade conditions in the Eyes-move task (top), and between retinotopic and spatiotopic conditions in the Eyes-move task (bottom). For each contrast, significant clusters in the positive direction are shown in green and negative in orange. Maps were cluster threshold corrected at p < 0.05. For spatiotopic > retinotopic contrast, the only significant cluster found was located in the left hemisphere, so only the left lateral viewing angle is shown here. Download Figure 6-3, TIF file. [file enu-eN-NWR-0186-20-s03.tif]

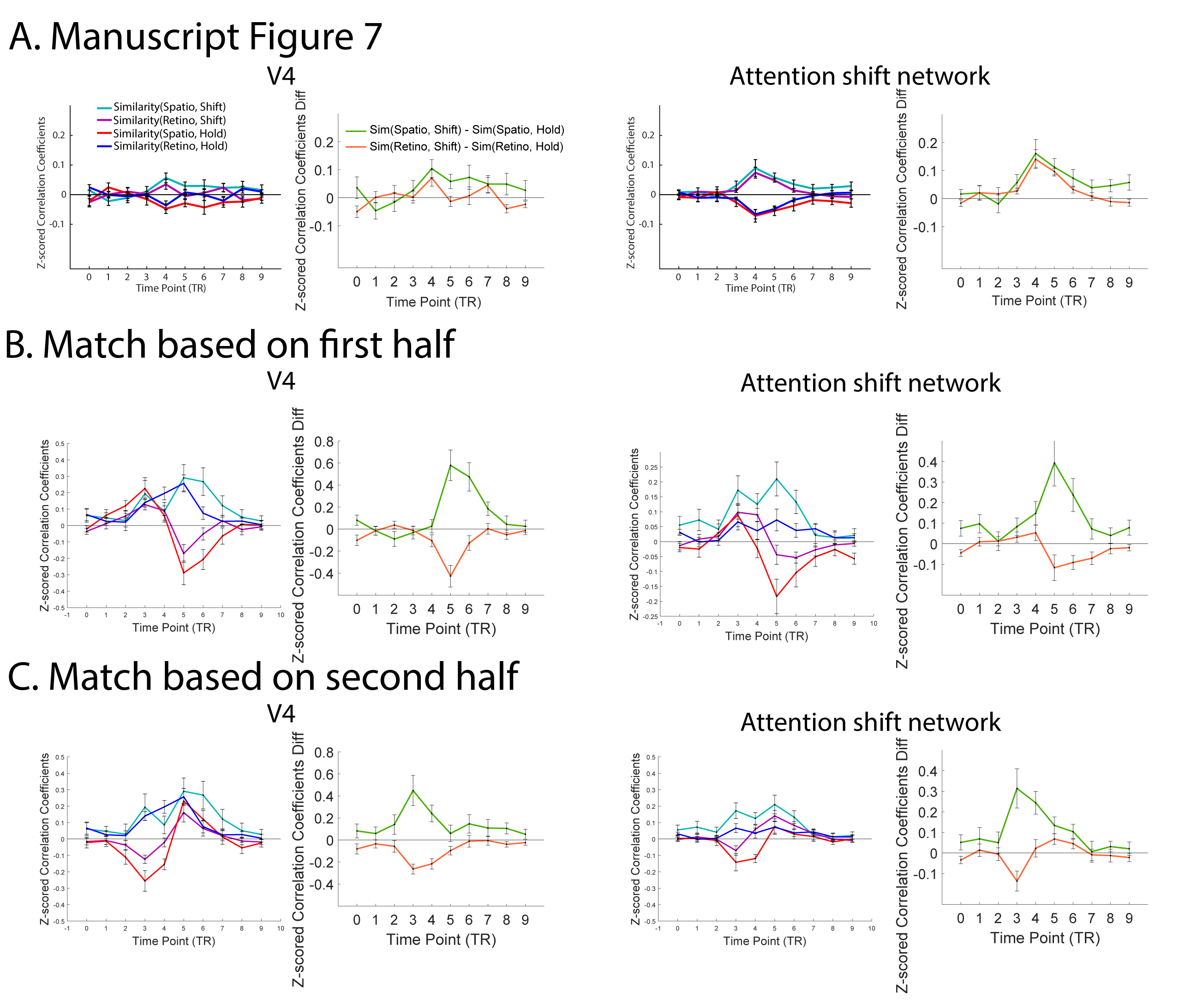

Supplement: Extended Data Figure 7-1 — Extended data showing an alternative way to analyze cross-task similarity, matching the hemispheric locations of covert attention, e.g., only correlating RetL and HoldL (RetR and HoldR) to calculate similarity between retinotopic and hold. Compared with the analysis in the paper (panel A), here, we perform this alternative analysis based on matching the first half of the trial (before shift/saccade; panel B) and matching the second half of the trial (after shift/saccade; panel C). In panel B, we can see that in both ROIs, at time point 3 when BOLD signals correspond to the first half of trial, all four pairs of correlations are positive, because we have explicitly matched the retinotopic location of attention for all. In the second half of the trial, now the correlations between retinotopic and hold (blue) and between spatiotopic and shift (cyan) are greater than those between retinotopic and shift (magenta) and spatiotopic and hold (red). This looks like the retinotopic attention condition has more representational similarity to holding attention, and the spatiotopic is more similar to shift, as reflected in the difference score plots. Panel C can be interpreted in a similar way. But again, this interpretation would be biased because we explicitly defined the conditions in terms of their retinotopic locations; thus, it is an unsurprising result. (Note, however, that the difference score plots are not symmetrical around zero, especially for the attention shift network; if the ROIs coded attention in a purely retinotopic manner, we would expect the difference curves to be of equal magnitude in opposite directions. Thus, even this retinotopically-biased analysis still reveals a pattern consistent with our original conclusions: that both retinotopic and spatiotopic attention in saccade trials carry some similarity to shifting attention.) Download Figure 7-1, TIF file. [file enu-eN-NWR-0186-20-s04.tif]

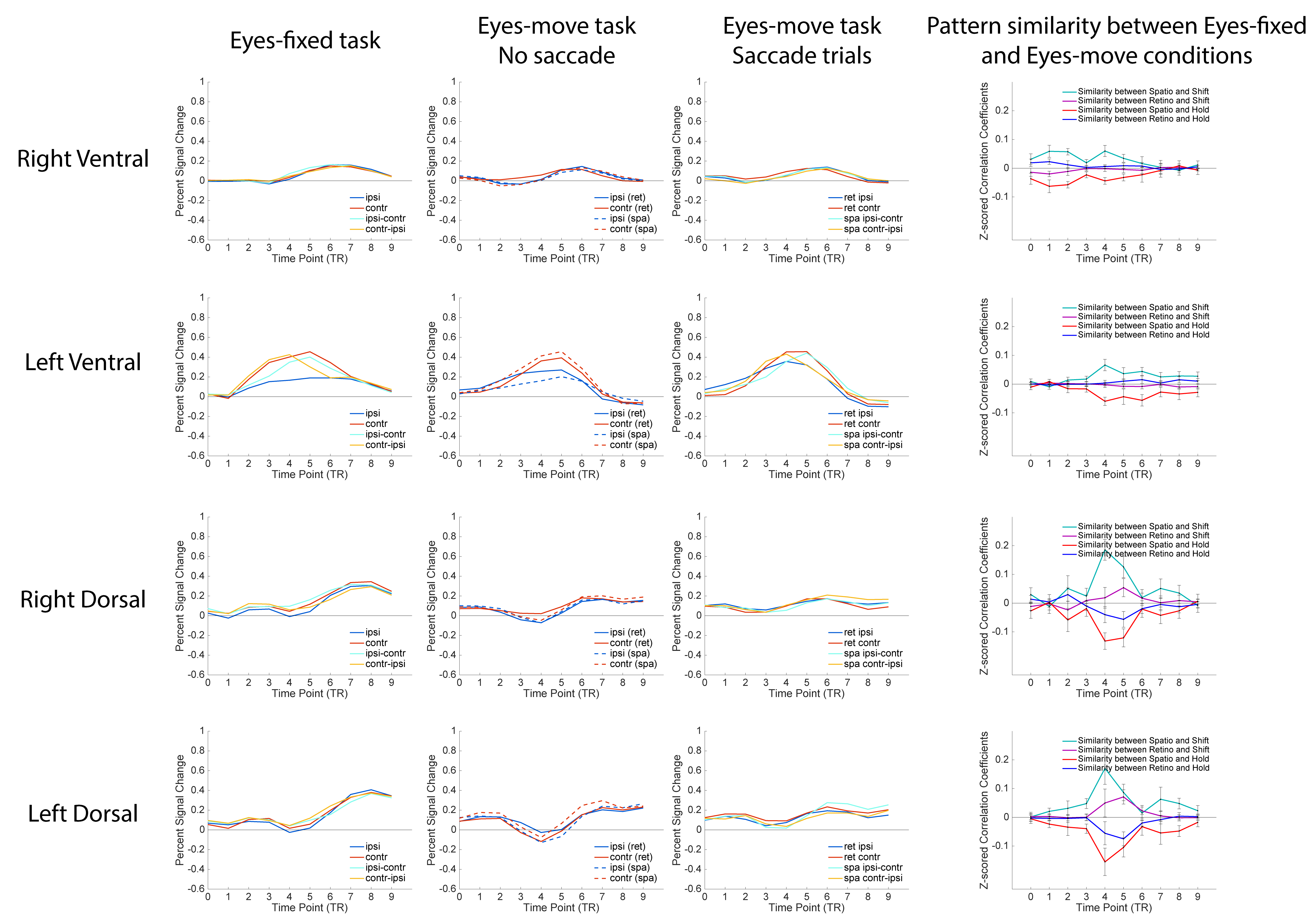

Supplement: Extended Data Figure 8-1 — Extended data showing univariate activation (the first three columns) and cross-task pattern similarities (the last column), separately for each cluster of the retinotopic-hold regions from the exploratory searchlight analyses. The univariate activation plots were comparable to Figure 4 and the pattern similarity plots to Figures 7C, 8B. Download Figure 8-1, TIF file. [file enu-eN-NWR-0186-20-s05.tif]
