# Supplementary material for: Neural Representations of Covert Attention across Saccades: Comparing Pattern Similarity to Shifting and Holding Attention during Fixation
Source: eNeuro. 2021 Mar 5;8(2):ENEURO.0186-20.2021. doi: 10.1523/ENEURO.0186-20.2021 (PMC8026251; doi:10.1523/ENEURO.0186-20.2021)
Supplement: Extended Data Table 3-1 — Statistics of 2×2 repeated-measure ANOVAs for V1 and task negative network at TP3, TP4, and TP5 respectively, on pattern similarity between Eyes-fixed conditions (hold & shift attention) and Eyes-move conditions (spatiotopic & retinotopic attention), separately for whole-trial analyses and time points of interest. Download Table 3-1, DOCX file. [file enu-eN-NWR-0186-20-s08.docx]

Table 3-1 Statistics of 2×2 repeated-measure ANOVAs for V1 and task negative network at TP3, TP4, and TP5 respectively, on pattern similarity between Eyes-fixed conditions (hold & shift attention) and Eyes-move conditions (spatiotopic & retinotopic attention), separately for whole-trial analyses and time points of interest.

|  | V1 | Task negative network |
| --- | --- | --- |
| Main effect of similarity to Eyes-fixed conditions  (hold & shift) | *F*=3.606, *p*=.084, *η_p_^2^*=.247  TP3: *F*=12.968, *p*=.004, *η_p_^2^*=.541**  TP4: *F*=0.575, *p*=.464, *η_p_^2^*=.050  TP5: *F*=1.469, *p*=.251, *η_p_^2^*=.118 | *F*=1.438, *p*=.256, *η_p_^2^*=.116  TP3: *F*=1.534, *p*=.241, *η_p_^2^*=.122  TP4: *F*=8.418, *p*=.014, *η_p_^2^*=.434**  TP5: *F*=9.492, *p*=.010, *η_p_^2^*=.463** |
| Main effect of Eyes-move conditions  (spatiotopic & retinotopic) | *F*=3.456, *p*=.090, *η_p_^2^*=.239  TP3: *F*=0.109, *p*=.748, *η_p_^2^*=.010  TP4: *F*=0.017, *p*=.898, *η_p_^2^*=.002  TP5: *F*=0.880, *p*=.368, *η_p_^2^*=.074 | *F*=0.397, *p*=.542, *η_p_^2^*=.035  TP3: *F*=0.012, *p*=.914, *η_p_^2^*=.001  TP4: *F*=4.807, *p*=.051, *η_p_^2^*=.304  TP5: *F*=3.332, *p*=.095, *η_p_^2^*=.232 |
| Interaction between similarity to Eyes-fixed conditions and similarity to Eyes-move conditions | *F*=0.473, *p*=.506, *η_p_^2^*=.041  TP3: *F*=0.232, *p*=.640, *η_p_^2^*=.021  TP4: *F*=0.229, *p*=.641, *η_p_^2^*=.020  TP5: *F*=0.026, *p*=.875, *η_p_^2^*=.002 | *F*=0.260, *p*=.620, *η_p_^2^*=.023  TP3: *F*=0.232, *p*=.639, *η_p_^2^*=.021  TP4: *F*=1.026, *p*=.333, *η_p_^2^*=.085  TP5: *F*=2.468, *p*=.144, *η_p_^2^*=.183 |

* indicate statistical significance at *p*<.05

** indicate statistical significance at *p*<.05 (Holm-Bonferroni corrected for multiple post hoc comparisons, separately across ROIs/networks for whole-trial beta weights, and across three TPs for time-course beta weights)
